# Antisense-mediated regulation of exon usage in the elastic spring region of Titin modulates sarcomere function

**DOI:** 10.1093/cvr/cvaf037

**Published:** 2025-03-05

**Authors:** Selvi Celik, Ludvig Hyrefelt, Tomasz Czuba, Yuan Li, Juliana Assis, Julia Martinez, Markus Johansson, Oscar André, Jane Synnergren, Joakim Sandstedt, Pontus Nordenfelt, Kristina Vukusic, J Gustav Smith, Olof Gidlöf

**Affiliations:** Division of Cardiology, Department of Clinical Sciences, Lund University, BMC D12, Solvegatan 19, Lund SE-221 84, Sweden; Wallenberg Centre for Molecular Medicine and Lund University Diabetes Centre, Lund University, Lund, Sweden; Division of Cardiology, Department of Clinical Sciences, Lund University, BMC D12, Solvegatan 19, Lund SE-221 84, Sweden; Division of Cardiology, Department of Clinical Sciences, Lund University, BMC D12, Solvegatan 19, Lund SE-221 84, Sweden; Wallenberg Centre for Molecular Medicine and Lund University Diabetes Centre, Lund University, Lund, Sweden; The Wallenberg Laboratory/Department of Molecular and Clinical Medicine, Institute of Medicine, Gothenburg University and The Department of Cardiology, Sahlgrenska University Hospital, Gothenburg, Sweden; Science for Life Laboratory, Gothenburg University, Gothenburg, Sweden; National Bioinformatics Infrastructure Sweden, Science for Life Laboratory, Lund University, Lund, Sweden; Department of Immunotechnology, Lund University, Lund, Sweden; National Bioinformatics Infrastructure Sweden, Science for Life Laboratory, Lund University, Lund, Sweden; Division of Cardiology, Department of Clinical Sciences, Lund University, BMC D12, Solvegatan 19, Lund SE-221 84, Sweden; Wallenberg Centre for Molecular Medicine and Lund University Diabetes Centre, Lund University, Lund, Sweden; Systems Biology Research Center, School of Bioscience, University of Skövde, SkövdeSweden; Division of Infection Medicine, Department of Clinical Sciences, Lund University, Lund, Sweden; Systems Biology Research Center, School of Bioscience, University of Skövde, SkövdeSweden; Department of Molecular and Clinical Medicine, Institute of Medicine, Sahlgrenska Academy, University of Gothenburg, Gothenburg, Sweden; Department of Laboratory Medicine, Institute of Biomedicine, Sahlgrenska Academy, University of Gothenburg, Gothenburg, Sweden; Division of Infection Medicine, Department of Clinical Sciences, Lund University, Lund, Sweden; Department of Laboratory Medicine, Institute of Biomedicine, Sahlgrenska Academy, University of Gothenburg, Gothenburg, Sweden; Division of Cardiology, Department of Clinical Sciences, Lund University, BMC D12, Solvegatan 19, Lund SE-221 84, Sweden; Wallenberg Centre for Molecular Medicine and Lund University Diabetes Centre, Lund University, Lund, Sweden; The Wallenberg Laboratory/Department of Molecular and Clinical Medicine, Institute of Medicine, Gothenburg University and The Department of Cardiology, Sahlgrenska University Hospital, Gothenburg, Sweden; Science for Life Laboratory, Gothenburg University, Gothenburg, Sweden; Division of Cardiology, Department of Clinical Sciences, Lund University, BMC D12, Solvegatan 19, Lund SE-221 84, Sweden; Wallenberg Centre for Molecular Medicine and Lund University Diabetes Centre, Lund University, Lund, Sweden

**Keywords:** Sarcomere function, Splicing, Non-coding RNA, Titin

## Abstract

**Aims:**

Alternative splicing of Titin (TTN) I-band exons produce protein isoforms with variable size and elasticity, but the mechanisms whereby TTN splice factors regulate exon usage and thereby determining cardiomyocyte passive stiffness and diastolic function, is not well understood. Non-coding RNA transcripts from the antisense strand of protein-coding genes have been shown to regulate alternative splicing of the sense gene. The *TTN* gene locus harbours >80 natural antisense transcripts (NATs) with unknown function in the human heart. The aim of this study was to determine if *TTN* antisense transcripts play a role in alternative splicing of *TTN*.

**Methods and results:**

RNA-sequencing and RNA *in situ* hybridization (ISH) of cardiac tissue from heart failure (HF) patients, unused donor hearts, and human iPS-derived cardiomyocytes (iPS-CMs) were used to determine the expression and localization of *TTN* NATs. Live cell imaging was used to analyse the effect of NATs on sarcomere properties. RNA ISH and immunofluorescence was performed in iPS-CMs to study the interaction between NATs, *TTN* mRNA, and splice factor protein RBM20. We found that *TTN-AS1-276* was the predominant *TTN* NAT in the human heart and that it was up-regulated in HF. Knockdown of *TTN-AS1-276* in human iPS-CMs resulted in decreased interaction between RBM20 and *TTN* pre-mRNA, decreased *TTN* I-band exon skipping, and markedly lower expression of the less compliant TTN isoform N2B. The effect on *TTN* exon usage was independent of sense–antisense exon overlap and polymerase II elongation rate. Furthermore, knockdown resulted in longer sarcomeres with preserved alignment, improved fractional shortening, and relaxation times.

**Conclusions:**

We demonstrate a role for *TTN-AS1-276* in facilitating alternative splicing of *TTN* and regulating sarcomere properties. This transcript could constitute a target for improving cardiac passive stiffness and diastolic function in conditions such as heart failure with preserved ejection fraction.

## Graphical Abstract

**Graphical Abstract:**
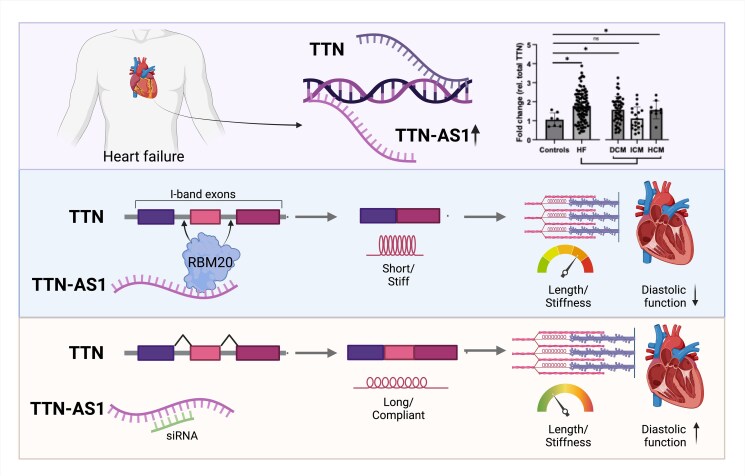

**Time of primary review: 39 days**

## 1. Introduction

Titin (TTN) is the largest protein in the human body (∼3 MDa) and constitutes a central component of the sarcomere.^1^ It is anchored in the Z-disc and extends to the M-band, aligned along the length of an entire half-sarcomere (physiological range in adult human cardiomyocytes: 1.8–2 µm).^2^ The presence of extensible PEVK-repeat and immunoglobulin-domains in the I-band region gives TTN elastic properties, acting as a biomechanical spring that contributes to sarcomere passive tension and provides elastic force in response to stretch.^3,4^ In the heart, TTN is a major determinant of myocardial passive stiffness^1,5^ and plays important roles in regulating diastolic function.^6–8^ Moreover, truncating genetic variants in *TTN* are the most common genetic lesion in patients with dilated cardiomyopathy (DCM),^9^ underscoring the importance of TTN in regulating contractile function. Elucidation of molecular mechanisms regulating TTN compliance could thus be of therapeutic importance for heart diseases characterized either by excessive myocardial stiffness and diastolic dysfunction, such as heart failure with preserved ejection fraction (HFpEF), or by systolic dysfunction, such as DCM.

The mechanical properties of TTN are primarily regulated at the post-transcriptional level, where a series of exon-skipping events among I-band exons produces an array of transcript isoforms differing in the number of extensible domains.^10^ Alternative splicing of the enormous 363 exon *TTN* gene is extraordinarily complex and presumably orchestrated through a highly regulated process involving a number of splicing factors. However, RNA binding motif protein 20 (RBM20) is currently the only protein known to promote alternative splicing of *TTN*.^11^ Consequently, pathogenic variants in *RBM20* have been implicated in 2–6% of DCM cases.^12^ RBM20 interacts with intronic motifs in pre-mRNA^13^ and prevents inclusion of up- and downstream exons through interference with U1 snRNP splice site recognition.^14^ However, mechanisms regulating recruitment of RBM20 to *TTN* pre-mRNA or additional factors affecting alternative splicing of TTN have not been elucidated.

Antisense transcription, i.e. transcription of non-coding RNA from the opposite strand of a coding gene, is pervasive throughout the human genome^15^ and can influence expression and splicing of the sense transcript. Morrissy *et al.*^16^ found a striking association between the presence of antisense transcription and alternative splicing of the sense gene across the human genome and postulated that slowing of the RNA polymerase II elongation rate at overlapping sense–antisense exons promotes alternative splicing of the sense transcript. Others have reported that antisense transcripts can mask splice sites in the sense transcript by forming an RNA-duplex with complementary sequences in the coding transcript^17^ or recruit specific components of the spliceosome to the sense gene pre-mRNA.^18,19^ The *TTN* locus spans >280 kb on chromosome 2q31 and harbours >80 annotated antisense transcripts, the function of which have not been studied in the heart previously.

The aims of this study were to map *TTN* antisense transcription in the human heart, to elucidate its possible role in regulation of *TTN* splicing, and to study the potential downstream implications of targeting specific *TTN* antisense transcripts on sarcomere organization and function.

## 2. Methods

A full description of methods can be found in Supplementary material online, *Methods*.

### 2.1 Human samples

Left ventricular biopsies from unused organ donor hearts (*n* = 7) and explanted cardiac tissue from heart failure patients (*n* = 100) were collected at transplantation centres at Lund University Hospital, Lund, Sweden and Sahlgrenska University Hospital, Gothenburg, Sweden, and stored at −80°C (patient characteristics are provided in *Table 1*). Informed consent was provided by patients or in the case of unused organ donor hearts, a close relative. The study was approved by the Ethics Board at Lund and Gothenburg University, respectively. The study was conducted in concordance with the Declaration of Helsinki. The methodology, conduct, and reporting of this study were in accordance with the Strengthening the Reporting of Observational Studies in Epidemiology (STROBE) statement for observational studies. STROBE recommendations for reporting observational studies are available as Supplementary material online, *Table S1*.

**Table 1:** Patient characteristics

	Non-failing controls	Heart failure patients
Sample source	Unused donor hearts	Explanted hearts
Number of individuals	7	100
Age, mean (SD), y	55.7 (12.3)	50.3 (14.1)
Sex		
Female (*n*)	3	25
Male (*n*)	4	75
Aetiology		
DCM, *n*		47
ICM, *n*		19
HCM, *n*		10
Other, *n*		24
Comorbidities		
Hypertension, *n*		17
Diabetes, *n*		9
Coronary artery disease, *n*		26
Stroke, *n*		14
Smoking		
Yes		0
Ex		38
No		62

### 2.2 Human heart muscle cells

Human iCell iPS-derived cardiomyocytes (iPS-CMs) were sourced from FujiFilm Cellular Dynamics Inc. (Madison, WI, USA) and Takara Bio (Takara Bio Europe, Saint-Germain-en-Laye, France). Cells were cultured as recommended by the manufacturers.

### 2.3 RNA isolation

Frozen tissue was cut into small pieces and homogenized in 700 μL of QIAzol (Qiagen, Hilden, Germany) using an Omni TH rotor-stator homogenizer. For isolation of RNA from cells, 700 μL of QIAzol was added directly to cell culture plates. For isolation of chromatin-enriched and soluble nuclear RNA fractions from cells, the protocol described by Werner *et al.*^20^ was used. Total RNA was isolated using the miRNeasy mini kit (Qiagen) according to the manufacturer’s instructions. The quantity and quality of isolated RNA was assessed with Qubit Flex (ThermoFisher) using the QuantIT RNA HS Assay Kit (ThermoFisher) and Agilent 4200 TapeStation (Agilent Technologies, Santa Clara, CA, USA) using the RNA ScreenTape Analysis Kit (Agilent Technologies).

### 2.4 siRNA and plasmid DNA transfection

For knockdown experiments, cells were transfected with Silencer Select siRNA (ThermoFisher) directed towards exon 1 (si276-Ex1, #n294437) or exon 12 (si276-Ex12, custom design ID #ABRSBMG) of TTN-AS1-276, towards *RBM20* (ENST00000369519.4, #s49081) or with a scrambled negative control siRNA sequence (#4390843). For visualization and tracking of sarcomeres, cells were transfected with a plasmid expressing *ACTN2* (*NM_001103.4*) with a eGFP tag.^21^ For immunoprecipitation of RBM20, cells were transfected with a pEZ-M03 vector (GeneCopoeia, Rockville, MD, USA) expressing RBM20 (ENST00000369519.4) with an eGFP tag. An in-frame deletion of a region spanning from exon 5 to exon 9 of the RBM20 open reading frame was made using site-directed mutagenesis (SDM) in order to produce a fusion protein lacking the RBM20 RNA recognition motif (RRM). SDM was carried out using the Phusion Site-Directed Mutagenesis Kit (ThermoFisher) according to the manufacturer’s instructions with forward primer 5′-GAGCCCAAAGCCAAGTCGGACAAGTAT-3′ and reverse primer 5′-CCTTGCTGGAATGGGCACGTATGATGTT-3′. Confirmation of the deletion was performed with PCR using the forward primer 5′-ATAACCCTGCTGGGAATGAAG-3′ and reverse primer 5′-CCACTGATTGAGGGCTTTCT-3′. Transfections were performed using Lipofectamine 3000 (ThermoFisher) according to the manufacturer’s instructions.

### 2.5 RNA *in situ* hybridization of human cardiac tissue

Human cardiac cryosections were fixed, dehydrated and pre-treated with Protease IV for the RNAScope Fluorescent Multiplex Assay (Advanced Cell Diagnostics, Hayward, CA, USA) according to the manufacturer’s recommendations. The RNAScope Multiplex Fluorescent Assay was performed using probes Hs-TTN-C1 (#550361, Advanced Cellular Diagnostics) and Hs-TTN-AS1-C2 (#1115141-C2, Advanced Cellular Diagnostics), according to the manufacturer’s recommendations. Before mounting, Wheat Germ Agglutinin-AlexaFluor488 (ThermoFisher) was added. Sections were then washed, counterstained with DAPI and mounted. Sections were imaged using a Nikon TiE TIRF microscope (Nikon Corporation, Tokyo, Japan) equipped with a Photometrics Prime95B sCMOS camera (Teledyne Photometrics, Tucson, AZ, USA).

### 2.6 Combined RNA *in situ* hybridization and immunofluorescence

iPS-CM was seeded in Lab-Tek 4-well chamber slides (Sigma-Aldrich) and fixed, de- and rehydrated and treated with Protease III in preparation for RNAScope Multiplex Fluorescent Assay (Advanced Cellular Diagnostics) according to the manufacturer’s instructions. The RNAScope Multiplex Fluorescent Assay was performed according to the manufacturer’s recommendations. Before mounting, immunofluorescent staining was performed with a rabbit anti-RBM20 antibody (Abcam, #ab233147) at 10 μg/mL for 1 h. An AlexaFluor488 anti-rabbit IgG secondary antibody (#4412 Cell Signaling, Danvers, MA, USA) at 1:1000 dilution was then added and incubated for 30 min. Slides were counterstained with DAPI and mounted. Slides were imaged using Operetta CLS high content screening instrument (PerkinElmer, Waltham, MA, USA), and the number and localization of *TTN* (Atto 550), *TTN-AS1* (Atto 647), and RBM20 (AlexaFluor488) fluorescent foci were analysed using Harmony 5.2 software.

### 2.7 Protein gel electrophoresis

Analysis of TTN protein isoforms was performed with agarose gel electrophoresis according to a previously established protocol.^22^ Protein bands representing TTN isoforms were visualized on ChemiDoc MP imaging system (Bio-Rad) and quantified using densiometric measurements in Image Lab 6.1 (Bio-Rad) and normalized to the myosin heavy chain 7 (MYH7) band.

### 2.8 RNA immunoprecipitation

Human iPS-CM was transfected with pCMV-RBM20-GFP plasmid DNA and siRNA as described elsewhere. RNA immunoprecipitation (RIP) was performed on 100 μL cell lysate per sample using the Magna RIP Kit (Merck) according to the manufacturer’s instructions with rabbit polyclonal anti-GFP antibody (#ab290, Abcam) or rabbit IgG antibody.

### 2.9 Chromatin immunoprecipitation

Preparation of cross-linked chromatin and chromatin immunoprecipitation was performed using the SimpleChIP Kit (Cell Signaling) according to the manufacturer’s instructions with ChIPAb+ anti-RNA Pol II mouse monoclonal antibody or mouse IgG antibody.

### 2.10 Sarcomere tracking

iPS-CM was transfected with pACTN2-GFP to visualize the sarcomere Z-discs. Live cell imaging of contracting iPS-CM was performed with wide-field epifluorescence microscopy using an ECLIPSE Ti2-E microscope (Nikon). Videos of contracting cells, capturing at least two contractions, were recorded at 30 frames per second using a Nikon DS-Qi2 CMOS camera. Segmentation of Z-discs and sarcomere tracking was then performed on a total of 32 video files in the SarcGraph software.^23^

### 2.11 qPCR and RT–PCR

cDNA was synthesized using the RevertAid First Strand cDNA Synthesis Kit (ThermoFisher) according to the manufacturer’s instructions and used in qPCR reactions with 2 × Universal TaqMan Master Mix (ThermoFisher) or in RT–PCR reactions with 2 × PCR Master Mix (ThermoFisher). The expression of *TTN*, *RBM20*, *GAPDH*, *CAMK2D*, *CACNA1C*, *LMO7*, *PRKRA*, *CCDC141*, *PLEKHA3*, *FKBP7*, and *DFNB59* was assessed with TaqMan Gene Expression Assays (ThermoFisher). For the quantification of specific splice products or exons from *TTN*, *TTN-AS1*, *CAMK2D*, *CACNA1C*, and *LMO7*, custom PrimeTime qPCR Probe Assays spanning specific exon–exon junctions or within exons were designed using the PrimerQuest Tool (Integrated DNA Technologies, Coralville, IA, USA). See Supplementary material online, *Table S2* for primer and probe sequences. All qPCR reactions were run on a Bio-Rad CFX 96 instrument (Bio-Rad, Hercules, CA, USA). For gene expression analysis, Ct-values were normalized to the reference gene *GAPDH* and expressed relative to the mean of the control group. For quantification of splicing products/isoforms, Ct-values were normalized to a qPCR assay designed to measure all transcripts from the corresponding gene and expressed relative to the mean of the control group. For quantification of immunoprecipitated RNA, Ct-values were transformed to ‘% of input’ using the Ct-value of the 2% Input sample with the formula 2^−(CtRIP − (CtInput − 3.32))^. RT–PCR reactions were run on an agarose gel with GelRed (Biotium, Fremont, CA, USA), and amplicons were visualized using a ChemiDoc MP imaging system with the ‘UV Trans’ application. Bands corresponding to the PCR amplicons were quantified using densiometric measurements in Image Lab 6.1 and normalized to the input samples.

### 2.12 RNA-sequencing

For human cardiac tissue samples, 800 ng of RNA was used as input for library preparation using the TruSeq Stranded Total RNA Library Prep Gold kit (Illumina, San Diego, CA, USA) with rRNA Removal Mix. Sequencing was performed on a NovaSeq 6000 system using the NovaSeq 6000 S4 reagent kit (Illumina) with 101 bp paired-end reads (see Supplementary material online, *Figure S1*).

For human iPS-CMs, 10 ng of RNA was used as input for cDNA synthesis using the SMART-Seq v4 Ultra Low Input RNA Kit for Sequencing (Takara Bio, San Jose, CA, USA). Library preparation was performed using the Nextera XT DNA Library Preparation Kit (Illumina). Sequencing was performed using the NovaSeq 6000 SP Reagent Kit v 1.0 (Illumina) with 101 bp paired-end reads. All mapped reads with mapping quality > 10 were counted analysed with DEXSeq 1.44.0.^24,25^ Per cent spliced in (PSI) was estimated using the Calculate-PSI package (https://github.com/jalwillcox/Calculate-PSI).

### 2.13 Statistical analysis

All data are shown with mean and standard deviation. Differences between experimental groups were assessed with Student’s *t*-test or ANOVA with Dunnett’s multiple comparisons test as specified in the figure legends. Correlation between continuous variables was assessed with linear regression. Associations between binary outcomes and continuous variables were assessed with binary logistic regression. All statistical analyses were performed in GraphPad Prism v. 10.

## 3. Results

### 3.1 Cardiac antisense transcription in the TTN locus

**Figure 1:**
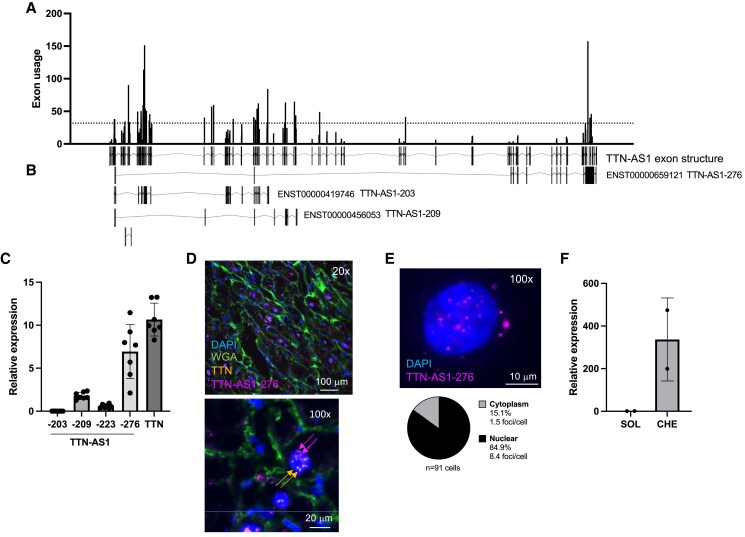
Expression and localization of *TTN* antisense transcripts in the human heart. (*A*) *TTN-AS1* exon usage was calculated using cardiac RNA-sequencing data from seven organ donor hearts without heart disease. The dashed line indicates the 75th percentile. (*B*) The *TTN-AS1* transcripts with the highest exon usage. (*C*) Relative expression of *TTN-AS1* transcripts and *TTN* with qRT–PCR in the seven organ donor hearts without heart disease. (*D*) RNA *in situ* hybridization (ISH) for *TTN-AS1-276* and *TTN* in a tissue section from one representative human cardiac biopsy. Cell membranes were stained with wheat germ agglutinin (WGA). (*E*) RNA ISH for *TTN-AS1-276* in human iPS-derived cardiomyocytes (iPS-CM). Nuclei were stained with DAPI. Below, quantification of the proportion of nuclear and cytoplasmic *TTN-AS1-276* ISH foci. (*F*) Relative expression of *TTN-AS1-276* in iPS-CM nuclear compartments with qRT–PCR. Data are derived from two different RNA preparations per group. SOL, soluble fraction; CHE, chromatin-enriched fraction.

Antisense transcription in the *TTN* gene locus is extensive, with 81 *TTN-AS1* transcripts annotated in GENCODE v. 44 (see Supplementary material online, *Figure S2A*). To obtain an overview of *TTN-AS1* transcript structure and expression in the human heart, we determined exon usage across all *TTN-AS1* isoforms by applying the DEXSeq analysis pipeline on RNA-sequencing data from left ventricular tissue without heart disease (*n* = 7, *Figure 1A*). Exon usage varied substantially within and between transcripts, but the high-expression (75th percentile) exons all belonged to a group of core TTN-AS1 transcripts (*Figure 1B*). We quantified the four TTN-AS1 transcripts with the highest expression exons (TTN-AS1-276, -209, -203, and -223) using the same human cardiac tissue without heart disease (*n* = 7) and custom qPCR assays targeting transcript-specific exons or exon–exon junctions (*Figure 1C*). The highest expression in cardiac tissue was observed for TTN-AS1-276 (ENST00000659121), which is also the ENSEMBL canonical transcript, defined as the single most representative and best-supported transcript from any given gene. TTN-AS1-276 is a 6297 bp transcript with 13 exons spanning >250 kb of the *TTN* gene (see Supplementary material online, *Figure S2A*). The transcriptional start site of *TTN-AS1*-276 lies ∼2500 bp downstream of *TTN* and a high-quality map of functional genomic regions based on Roadmap Epigenomics data^26^ showed that this genomic region is an active promoter in human atrial and ventricular tissue (see Supplementary material online, *Figure S2B*). RNA *in situ* hybridization (ISH) revealed widespread expression of *TTN-AS1-276* in human left ventricular tissue (*Figure 1D* and Supplementary material online, *Figure S3*), localized predominantly in cardiomyocytes (as defined by large cell/nuclear size and presence of *TTN* ISH foci). Cardiomyocyte-specific expression of *TTN-AS1* was corroborated by GTEx cardiac single nucleus RNA-sequencing data,^27^ where median log-normalized and scaled expression counts for *TTN-AS1* were >10 000-fold higher in cardiomyocytes than in any other cardiac cell type.

Clues regarding the functional role of a natural antisense transcript can be drawn from its intracellular localization.^28^ We therefore performed RNA ISH on human iPS-CMs and observed that ∼85% of *TTN-AS1-276* foci were localized in the nucleus (*Figure 1E*). Moreover, fractionation of iPS-CM nuclear RNA revealed ∼300-fold higher levels of *TTN-AS1-276* in the chromatin compartment over the soluble compartment (*Figure 1F*). We conclude that *TTN-AS1-276* is the predominant *TTN* antisense transcript in the human heart and that it is localized to cardiomyocyte nuclear chromatin.

### 3.2 Design and validation of siRNAs targeting TTN-AS1-276

In order to study the functional role of *TTN-AS1-276*, we designed two independent siRNAs directed towards exons 1 and 12 of *TTN-AS1-276* (si276-Ex1 and si276-Ex12). Both si276-Ex1 and si276-Ex12 caused a statistically significant ∼40% decrease in *TTN-AS1-276* expression (see Supplementary material online, *Figure S4A*) but the expression of the other main *TTN-AS1* isoforms (-203, -209, and -223) was unaffected (see Supplementary material online, *Figure S4A*), confirming the efficacy and specificity of these siRNAs.

### 3.3 TTN-AS1-276 does not regulate TTN expression

Natural antisense transcripts often play a role as cis-acting transcriptional regulators of the sense protein-coding gene.^29^ To assess whether *TTN-AS1-276* affects the expression of *TTN*, we transfected human iPS-CM with si276-Ex1 and si276-Ex12 but detected no effect on expression of *TTN* or any of the other protein-coding genes immediately within 100 kb up- and downstream of *TTN* (see Supplementary material online, *Figure S4B*). We conclude that *TTN-AS1* does not play a role as a cis-acting transcriptional regulator.

### 3.4 TTN-AS1-276 regulates splicing of TTN I-band exons

**Figure 2:**
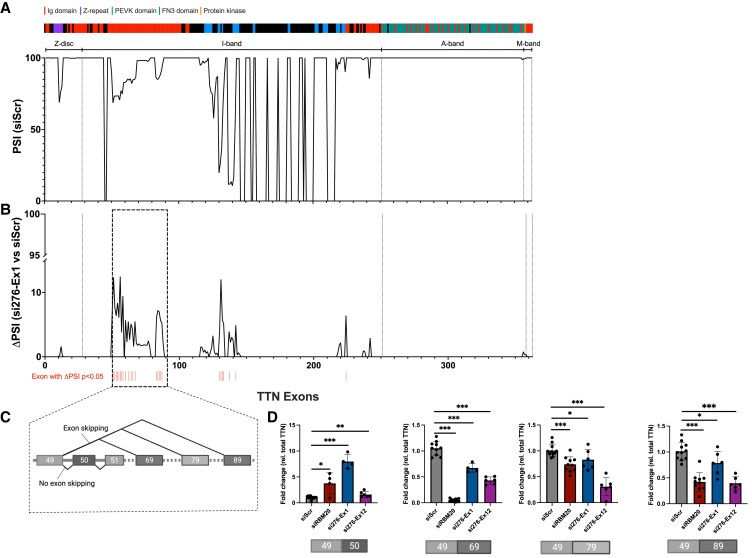
*TTN-AS1-276* regulates inclusion of I-band exons in *TTN*. (*A*) Per cent spliced in (PSI) for all exons across the *TTN* gene calculated based on RNA-sequencing reads from human iPS-derived cardiomyocytes (IPS-CM). The line represents the mean of three replicates (individual RNA preparations) per experimental group. (*B*) The difference in PSI (ΔPSI) comparing cells transfected with siRNA to *TTN-AS1-276* (si276-Ex1) with cells transfected with scrambled negative control siRNA (siScr). Exons with statistically significant ΔPSI are marked with red bars (adjusted *P* < 0.05). (*C*) Depiction of exon skipping from *TTN* exon 49. (*D*) Quantification of *TTN* splice products in iPS-CM transfected with si276-Ex1, si276-Ex12, siRBM20 or siScr, using custom qRT–PCR assays spanning the indicated exon–exon junctions. Expression data are normalized to that of total *TTN* and expressed relative to the mean of the negative control cells (siScr). Data are derived from two separate experiments with 3–6 replicates (individual RNA preparations) per experimental group. Differences between each individual experimental group and the control group were assessed with Student’s *t*-tests, *P < 0.05, **P < 0.01, ***P < 0.001.

We next hypothesized that *TTN-AS1* could influence alternative splicing of *TTN*, as the presence of antisense transcription across the human genome has been shown to associate strongly with alternative splicing of the corresponding sense gene.^16^ We performed RNA-seq on human iPS-CM transfected with si276-Ex1 or, as a positive control, siRNA towards *RBM20* (siRBM20), a splicing factor that mediates exon skipping in TTN,^13^ and calculated proportion spliced-in (PSI) for each *TTN* exon. Successful knockdown of *TTN-AS1-276* and *RBM20* was confirmed with qPCR (see Supplementary material online, *Figure S5A*). *TTN* PSI in control iPS-CM correlated well (Pearson *r* = 0.837, *P* < 0.0001) with previously reported data from human left ventricular tissue,^30^ with extensive splicing out of exons across the I-band region throughout exons 50–242 (numbered according to the complete inferred TTN meta-transcript, Supplementary material online, *Figure S5B*, *Figure 2A*). Knockdown of *TTN-AS1-276* caused significantly increased PSI for 26 exons (indicated by red bars in *Figure 2B*). Affected exons were all situated in the I-band and had baseline PSI values in the range of 70–85%. The most pronounced effect of *TTN-AS1-276* knockdown was observed in exons immediately downstream of exon 49 (exons 50–89, indicated by a dashed square in *Figure 2B*). These exons are normally spliced out of *TTN* through RBM20-mediated exon skipping^31^ and alternative splicing/exon skipping from exon 49 produces a range of *TTN* splice products,^32^ notable examples of which are shown in *Figure 2C*. Knockdown of *RBM20* resulted in a similar increase in PSI in exons downstream of exon 49 (see Supplementary material online, *Figure S5C*), confirming its role in mediating I-band exon skipping. To study whether the increased PSI in exons downstream of 49 following TTN-AS1 knockdown was caused by decreased exon skipping, we quantified the primary splice products across exon 49 splice junctions in iPS-CM transfected with si276-Ex1, si276-Ex12, or siRBM20 using qPCR. We observed a significant decrease in expression of *TTN* splice products dependent on exon skipping and a reciprocal increase in the isoform where exon 49 is spliced directly with exon 50 (*Figure 2D*). Considering the heterogeneity of iPS-CM, we validated the results using another iPS-CM cell line (see Supplementary material online, *Figure S6A*). We conclude that *TTN-AS1-276* regulates exon usage in I-band *TTN* through facilitating exon skipping downstream of exon 49.

### 3.5 TTN-AS1-276 regulates TTN isoform composition

**Figure 3:**
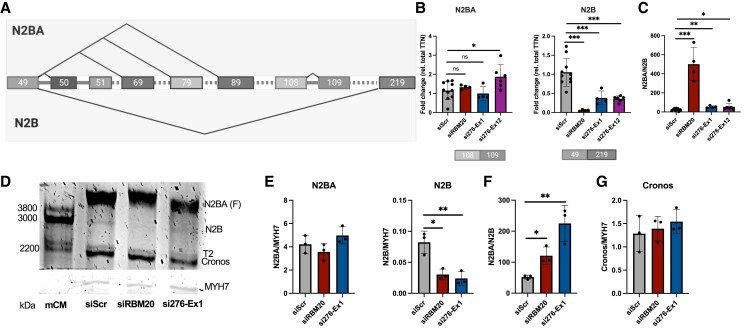
*TTN-AS1-276* knockdown causes a shift in TTN isoform composition. (*A*) Depiction of alternative splicing pathways from *TTN* exon 49 producing the two major cardiac isoforms, N2BA and N2B. (*B*) Quantification of N2BA and N2B isoforms in iPS-CM transfected with si276-Ex1, si276-Ex12, siRBM20, or siScr, using custom qRT–PCR assays spanning the indicated exon–exon junctions. Expression data are normalized to that of total *TTN* and expressed relative to the mean of the negative control cells (siScr). Data are derived from two separate experiments with two replicates (individual RNA preparations) per experimental group, ***P < 0.001. (*C*) The N2BA to N2B ratio in iPS-CM following transfection with si276-Ex1, si276-Ex12, siRBM20, or siScr, derived from data in (*B*). (*D*) Gel electrophoresis of protein from iPS-CM transfected with siScr, siRBM20, or siTTN-AS1. A sample of mouse cardiac protein (mCM) was run in one lane as a molecular ruler. Molecular weights of the major TTN isoforms (N2BA, N2B, and Cronos) are indicated. T2, TTN degradation product. The band corresponding to MYH7 is shown in the bottom. (*E* and *G*) Quantification of band intensity for N2BA, N2B, and Cronos relative to MYH7. (*F*) Ratio of N2BA to N2B. Data are derived from three separate experiments. Differences between each individual experimental group and the control group were assessed with Student’s *t*-tests *P < 0.05, **P < 0.01.

Cardiac *TTN* is composed of two primary isoforms, N2BA and N2B, differing in the extent to which I-band domains are included. N2B is produced by splicing of exon 49 with exon 219, excluding many extensible I-band domains and resulting in a short and less elastic TTN protein isoform (*Figure 3A*). As our data showed that *TTN-AS1-276* promotes exon skipping downstream of exon 49, we hypothesized that knockdown of *TTN-AS1* would result in decreased expression of the N2B isoform. To address this, we quantified expression of N2B and N2BA in iPS-CM transfected with si276-Ex1, si276-Ex12, or siRBM20 using custom qPCR assays spanning the exon 49–219 junction (N2B) and the exon 108–109 junction (constitutively included in N2BA). We observed significantly lower expression of the N2B isoform in iPS-CM where *TTN-AS1-276* had been knocked down, whereas expression of N2BA was unaffected (*Figure 3B*). This resulted in a two-fold increase in the N2BA:N2B ratio (*Figure 3C*). We confirmed these results in a separate iPS-CM line (see Supplementary material online, *Figure S6B*). Knockdown of *RBM20* caused a dramatic decrease in N2B expression and a pronounced increase in the N2BA:N2B ratio, in line with previous reports.^11,13^ Next, we analysed the consequence of *TTN-AS1-276* knockdown on N2B and N2BA protein isoforms using gel electrophoresis. As previously reported,^30^ iPS-CM expressed a longer, foetal-like N2BA isoform and N2B expression was considerably lower than in adult cardiac tissue (*Figure 3D*). Nevertheless, in line with our observation on the mRNA level, we found N2B protein expression to be significantly lower in iPS-CM where *TTN-AS1-276* had been knocked down but saw no effect on N2BA expression. This was also reflected in a significantly increased N2BA:N2B ratio. Again, the effect of *RBM20* knockdown had a similar effect on N2B expression and the N2BA:N2B ratio (*Figure 3F*). The Cronos TTN isoform, which is transcribed from an internal promoter in the distal I-band region (between exons 239 and 240 of the inferred meta-transcript), was neither affected by knockdown of *TTN-AS1-276* nor *RBM20* (*Figure 3G*). We conclude that *TTN-AS1-276* regulates *TTN* isoform composition and can be targeted to promote translation of longer and more extensible TTN.

### 3.6 TTN-AS1-276 regulates sarcomere function and cardiomyocyte contraction dynamics

**Figure 4:**
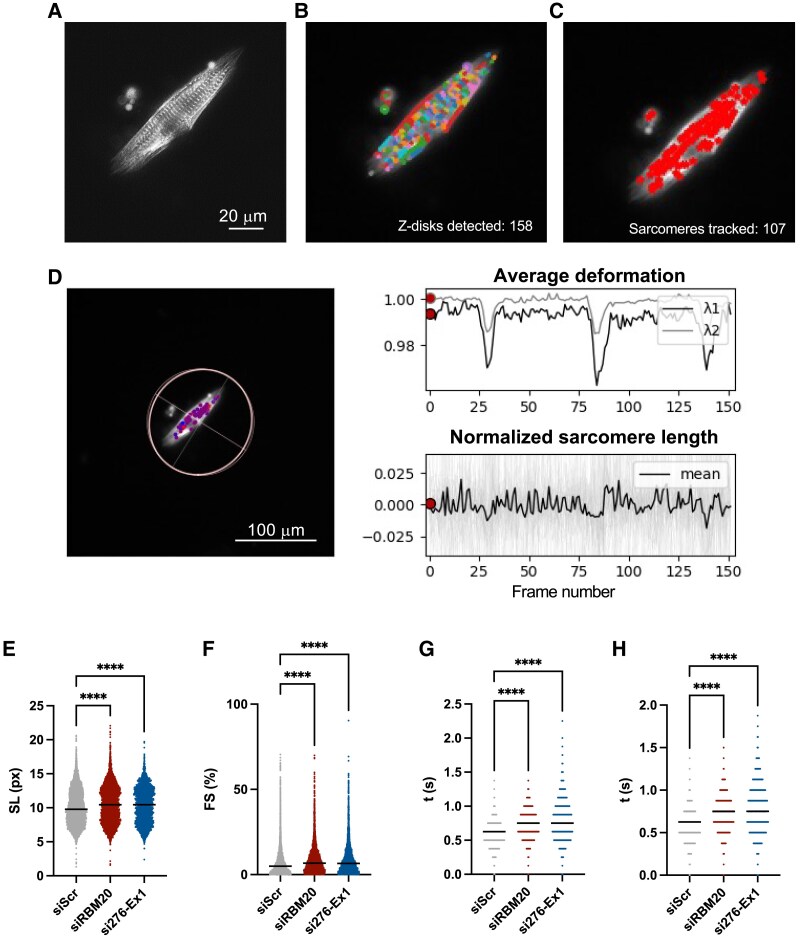
*TTN-AS1* knockdown results in increased sarcomere length and altered contraction dynamics. (*A*) Representative fluorescent image of a human iPS-derived cardiomyocyte transfected with a ACTN2-GFP plasmid for visualization and tracking of sarcomeres in SarcGraph software. Visualization of (*B*) Z-disc and (*C*) sarcomere segmentation in SarcGraph software. (*D*) Time series data for average deformation and normalized sarcomere length during three contractions. (*E*) Sarcomere length (SL), (*F*) fractional shortening (FS), (*G*) contraction time, and (*H*) relaxation time from a total of 14 636 segmented iPS-CM sarcomeres following transfection with si276-Ex1, siRBM20, or siScr. Data are derived from two separate experiments. Differences between each individual experimental group and the control group were assessed with Student’s *t*-tests, ****P < 0.0001.

TTN isoform composition is a key determinant of cardiomyocyte mechanical properties. As knockdown of *TTN-AS1-276* resulted in increased inclusion of I-band exons and a shift towards longer and more elastic TTN, we wanted to investigate the consequences of *TTN-AS1-276* knockdown on sarcomere structure and dynamics. To this end, we applied sarcomere tracking analysis on live iPS-CM after transfection with si276-Ex1 or siRBM20. Cells were transfected with a plasmid encoding GFP-tagged alpha-actinin-2 (pACTN2-GFP) to visualize sarcomere Z-discs (*Figure 4A*) and recorded during multiple contractions with live cell imaging (see Supplementary material online, *Video S1*). SarcGraph v.0.2.1 was used to detect, track, and analyse functional parameters of 14 636 sarcomeres from >200 cells during contraction (*Figure 4B–H*). Results showed that sarcomere length was increased by ∼10% (*P* < 0.001) in cells where *TTN-AS1-276* had been knocked down (*Figure 4E*), whereas the sarcomere alignment, as measured by the orientational order parameter, was unaffected (see Supplementary material online, *Figure S7*). Expectedly, knockdown of *RBM20* caused a similar increase in sarcomere length (*P* < 0.001). Fractional shortening (FS) was increased from 7.5% in control cells to 9% in cells transfected with si276-Ex1 (*P* < 0.001), indicating increased contractility (*Figure 4F*) and both mean contraction (*Figure 4G*) and mean relaxation (*Figure 4H*) time increased by ∼30–40% (*P* < 0.001) following *TTN-AS1-276* knockdown. Knockdown of *RBM20* mirrored these effects as well. We conclude that knockdown of *TTN-AS1-276* results in longer and more compliant sarcomeres with improved contractile properties.

### 3.7 No impact of overlapping antisense transcript exons on TTN exon usage and Pol II elongation rate

Next, we wanted to explore the mechanism by which *TTN-AS1* affects alternative splicing of *TTN*. Morrissy *et al.*^16^ hypothesized that a reason for the increased alternative splicing observed in exons with overlapping antisense exons is that polymerase elongation rates are decreased, as slower RNA polymerase II (Pol II) rates have been shown to increase the rate of alternative splicing.^33^ When considering exons from all annotated *TTN-AS1* and *TTN* transcript isoforms in GENCODE, ∼16% of *TTN* exons overlap with one or more *TTN-AS1* exons (red bars in Supplementary material online, *Figure S8A*). Apart from the Z-disc, overlapping exons are present throughout the whole *TTN* gene. If the presence of an antisense exon was to promote exon skipping in *TTN*, we expected usage of such *TTN* exons to be lower compared to those without an antisense exon. To analyse this, we leveraged DEXSeq exon usage data from human left ventricular tissue without heart disease (*n* = 7), but the results revealed instead a significantly increased usage of TTN exons with an overlapping antisense exon (see Supplementary material online, *Figure S8A* and *B*), contradicting this hypothesis. Moreover, we observed no enrichment of exons with an overlapping antisense exon among the 26 *TTN* exons with increased PSI following *TTN-AS1* knockdown (12% vs. 16% in *TTN* overall).

We next investigated Pol II occupancy across a selection of *TTN* exons with and without *TTN-AS1* exon overlap in iPS-CM using ChIP-PCR. We designed primers for six pairs of adjacent *TTN* exons where one exon had an overlapping antisense exon and the other did not (see Supplementary material online, *Figure S8C*), across different TTN domains and reflecting varying PSIs (see Supplementary material online, *Figure S8D*). With the exception of exon 48, for which no Pol II signal could be detected, there was no robust or meaningful difference in Pol II occupancy at exons with overlapping antisense exons compared to adjacent exons without overlapping antisense exons (see Supplementary material online, *Figure S8D*). Moreover, inhibition of Pol II elongation by addition of DRB did not affect the degree of *TTN-AS1* chromatin enrichment in iPS-CM, indicating that Pol II pausing does not influence *TTN-AS1* tethering to chromatin (see Supplementary material online, *Figure S8E*). Taken together, these data show that *TTN-AS1* influences *TTN* exon usage/alternative splicing in a manner independent of specific sense–antisense exon overlap and Pol II elongation rate.

### 3.8 TTN-AS1 facilitates interaction between RBM20 and TTN mRNA

**Figure 5:**
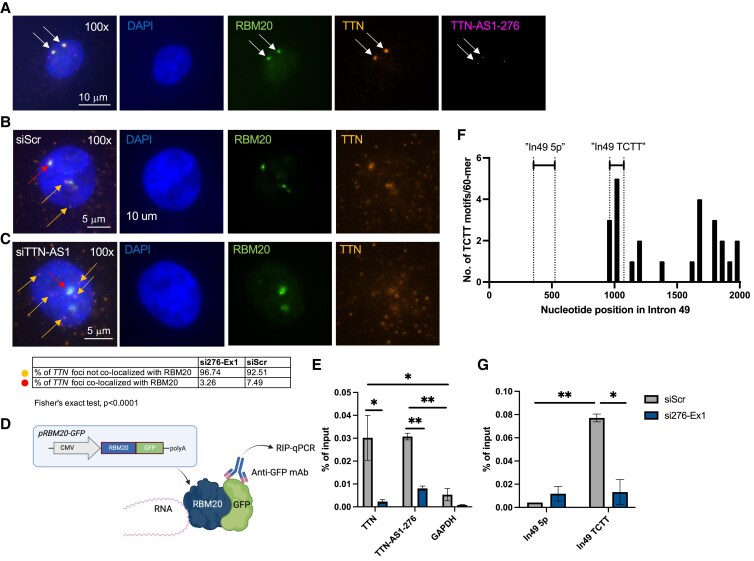
*TTN-AS1-276* facilitates interaction between RBM20 and *TTN* mRNA. (*A*–*C*) Human iPS-derived cardiomyocytes (iPS-CM) were subjected to combined RNA *in situ* hybridization (ISH) for *TTN-AS1-276* (magenta) and *TTN* (orange) and immunofluorescence for RBM20 (green) following transfection with siRNA towards *TTN-AS1-276* (si276-Ex1), *RBM20* (siRBM20), or scrambled negative control siRNA (siScr). Nuclei were counterstained with DAPI. Co-localization of *TTN* and RBM20 foci was analysed with high content imaging. (*D*) Depiction of experimental design for the RBM20 RNA immunoprecipitation (RIP) experiment. iPS-CM was transfected with a plasmid expressing a RBM20-GFP fusion protein. RIP was performed on iPS-CM protein using a GFP antibody and qRT–PCR was used to analyse immunoprecipitated RNA. (*E*) Enrichment of *TTN* and *TTN-AS1-276* in GFP-RBM20 RIP RNA from iPS-CM transfected with si276-Ex1 or siScr, analysed with qRT–PCR. Analysis of unrelated *GAPDH* RNA was included as a negative control. Data are derived from two separate experiments with two technical replicates (individual immunoprecipitates) in each group *P < 0.05, **P < 0.01. (*F*) The number of TCTT motifs/60 bp across intron 49 of *TTN*. The positions of RIP-qPCR assays used in (*G*) are indicated with dashed lines. (*G*) qPCR of GFP-RBM20 RIP RNA from (*E*) using assays targeting regions in intron 49 without TCTT motifs (‘In49 5p’) and enriched with TCTT motifs (‘In49 TCTT’). Data are derived from two separate experiments with two technical replicates (individual immunoprecipitates) in each group. Differences in the RIP signal between cells transfected within and between groups were assessed using ANOVA with Dunnett’s multiple comparisons test, *P < 0.05, **P < 0.01.

We then wanted to explore alternative mechanisms whereby *TTN-AS1* could regulate alternative splicing of *TTN*. Antisense transcripts have previously been reported to influence splicing either by forming an RNA-duplex with complementary sequences in the coding transcript, thereby masking splice sites in pre-mRNA of the sense gene^17^ or by recruiting or guiding specific components of the spliceosome to the sense gene pre-mRNA.^18,19^ We reasoned that *TTN-AS1-276* could exert its function through either of these mechanisms, but given the similar patterns in *TTN* ΔPSI, *TTN* isoform composition and functional consequences on sarcomere dynamics in iPS-CM following knockdown of *TTN-AS1-276* and *RBM20*, we believed that a mechanism whereby *TTN-AS1-276* recruits RBM20 to *TTN* mRNA would be more plausible. To explore these hypotheses, we analysed the quantity and nuclear localization of *TTN-AS1*, *TTN* mRNA, and RBM20 protein in iPS-CM using combined RNA ISH and immunofluorescence. First, we found that nuclear *TTN*:*TTN-AS1* co-localization, indicative of duplex formation, was rare, occurring once in every fifth cell on average, and interestingly, was then almost exclusively (>90% of instances) observed as part of a cluster involving RBM20 (*Figure 5A* and Supplementary material online, *Figure S9A*), with a fluorescence pattern indicative of RBM20-mediated *TTN* splicing.^31,34^ This observation contradicts a mechanism involving direct TTN:TTN-AS1 duplex formation and strengthens the hypothesis that *TTN-AS1* regulates *TTN* splicing via RBM20. To test this hypothesis further, we quantified the proportion of *TTN* RNA ISH foci co-localized with RBM20 in iPS-CM transfected with si276-Ex1 using high content imaging analysis. In control cells, we estimated that ∼7.5% of all nuclear *TTN* RNA ISH foci co-localized with RBM20 (*Figure 5B*). Interestingly, in iPS-CM where *TTN-AS1-276* had been knocked down, the proportion of *TTN* foci co-localized with RBM20 was more than halved (*P* < 0.0001, *Figure 5C*). This suggests that *TTN-AS1-276* facilitates interaction between RBM20 and *TTN* pre-mRNA. We then sought to corroborate these findings using RIP on iPS-CM. iPS-CM was transfected with a plasmid encoding a RBM20-GFP fusion protein (pRBM20-GFP), and a GFP antibody was then used to pull down the RBM20 RNA interactome (*Figure 5D*). Transfection with pRBM20-GFP resulted in transgenic expression of RBM20 fusion protein (see Supplementary material online, *Figure S10A*). We observed significant enrichment of both *TTN* and *TTN-AS1-276* in RBM20-RIP RNA compared to unrelated *GAPDH* RNA (*Figure 5E*) and to negative control IgG IP (see Supplementary material online, *Figure S10B*). Expectedly, the *TTN-AS1-276* RIP signal was significantly reduced in cells where *TTN-AS1-276* had been knocked down. Interestingly, there was also significantly less *TTN* interacting with RBM20 after *TTN-AS1-276* knockdown, giving additional support for a mechanism whereby *TTN-AS1* facilitates interaction between RBM20 and *TTN* mRNA (*Figure 5E*).

RBM20 mediates exon skipping by interacting with intronic TCTT motifs in pre-mRNA.^13^ We hypothesized that *TTN-AS1-276* assists in the binding of RBM20 to such intronic motifs upstream of skipped exons. To test this hypothesis, we chose to focus on intron 49, with which RBM20 would interact for skipping of exon 50 to occur. We scanned the sequence of intron 49 and found an enrichment of TCTT motifs in the middle of the intron and towards the 3′ end, whereas the 5′ end of the intron was completely devoid of TCTT motifs (*Figure 5F*). To assess binding of RBM20 to different regions of intron 49, we designed qPCR assays targeting a 5′ region free from TCTT motifs (‘In49 5p’) and a region enriched in TCTT motifs in the middle of the intron (‘In49 TCTT’). We then performed RBM20-RIP qPCR using these assays and expectedly, found that RBM20 was significantly enriched in the TCTT-rich region (*Figure 5G*). Interestingly, RBM20 binding to the TCTT-rich region was significantly reduced upon knockdown of *TTN-AS1-276* (*Figure 5G*). Taken together, these results point towards a mechanism whereby *TTN-AS1-276* mediates exon skipping in *TTN* through interacting with and recruiting RBM20 to intronic TCTT motifs. We then sought to explore the mode-of-action for the protein–RNA interaction and hypothesized that RBM20 could bind directly to *TTN-AS1-276* through its RRM. To test this, we performed an in-frame deletion of the RRM-coding exons in the pRBM20-GFP construct using SDM (see Supplementary material online, *Figure S11A*). We then transfected iPS-CM with the resulting plasmid (pRBM20ΔRRM-GFP) and performed RIP. We observed a near-complete loss of the *TTN-AS1-276* RIP signal in cells transfected with pRBM20ΔRRM-GFP (see Supplementary material online, *Figure S11B*), suggesting that RBM20 binds *TTN-AS1-276* directly through the RRM domain.

### 3.9 TTN-AS1 mediates splicing of other RBM20 targets

**Figure 6:**
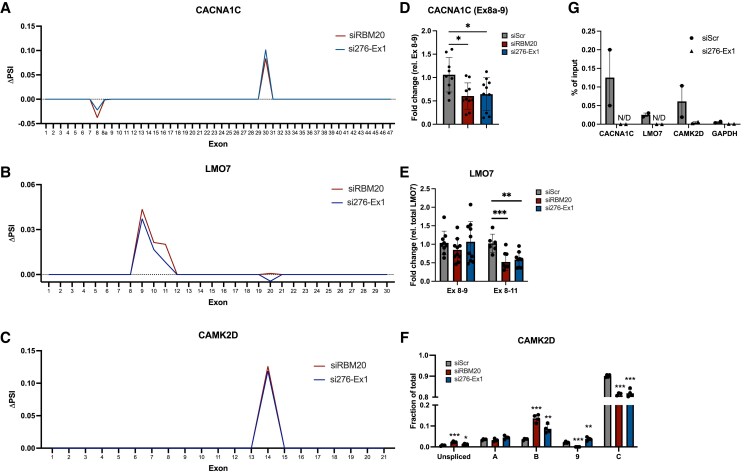
*TTN-AS1-276* facilitates splicing of additional RBM20 targets. (*A*–*C*) Difference in PSI (ΔPSI) comparing cells transfected with siRNA to *TTN-AS1-276* (si276-Ex1, blue) or *RBM20* (siRBM20, red) with cells transfected with scrambled negative control siRNA (siScr) across all exons of the (*A*) *CACNA1C*, (*B*) *LMO7*, and (*C*) *CAMK2D* genes in human iPS-derived cardiomyocytes (iPS-CM). The lines represent the mean of three technical replicates (individual RNA preparations). (*D*–*F*) Relative expression of alternative splice products of the (*D*) *CACNA1C*, (*E*) *LMO7*, and (*F*) *CAMK2D* genes in iPS-CM transfected with siRBM20, si276-Ex1 or siScr and analysed with qRT–PCR (*CACNA1C* and *LMO7*) and semi-quantitative RT–PCR (*CAMK2D*), respectively. Data are derived from three separate experiments with three technical replicates (individual RNA preparations) in each group. Differences between each individual experimental group and the control group were assessed with Student’s *t*-tests, *P < 0.05, **P < 0.01, ***P < 0.001. (*G*) Enrichment of *CACNA1C*, *LMO7*, and *CAMK2D* in GFP-RBM20 RIP RNA from iPS-CM transfected with si276-Ex1 or siScr, analysed with qRT–PCR. Analysis of unrelated *GAPDH* RNA was included as a negative control. Data are derived from two separate experiments.

In a previous study, Bertero *et al.*^34^ postulated that RBM20 forms a *trans-*interacting chromatin domain (TID), driving spatial proximity of multiple genomic loci representing a network of different RBM20 targets. The assembly of this splicing complex is initiated at the *TTN* genomic locus, where transcription of *TTN* nucleates RBM20 foci, which drives formation of the TID. Given the overlap and spatial proximity of *TTN-AS1-276* and *TTN* loci, the evidence of physical interaction of *TTN-AS1-276* and *TTN* RNA with RBM20 protein and the observation that the number of RBM20 protein foci decreased in iPS-CM after *TTN-AS1-276* knockdown (see Supplementary material online, *Figure S9B*), we hypothesized that *TTN-AS1-276* might facilitate the assembly of the RBM20 splicing factory. If this was the case, knockdown of *TTN-AS1-276* would affect splicing of not just *TTN*, but also of other RBM20 targets. To explore this hypothesis, we first analysed PSI data from iPS-CM where either *TTN-AS1-276* or *RBM20* had been knocked down, focusing on three genes shown to be included in the RBM20 TID: *CACNA1C*, *CAMK2D*, and *LMO7* (see Supplementary material online, *Figure S12A*). In line with previous reports, there was significantly different ΔPSI for exons 8 and 30 in *CACNA1C* (*Figure 6A*), exons 9–11 in LMO7 (*Figure 6B*), and exon 14 in *CAMK2D* (*Figure 6C*) following RBM20 knockdown. Interestingly, this effect was mirrored almost exactly in cells where *TTN-AS1-276* had been knocked down. Guided by these results, we quantified splicing products using qPCR assays spanning isoform-specific exon–exon junctions (*CACNA1C* and *LMO7*) or with RT–PCR (*CAMK2D*). For *CACNA1C*, we saw a significant down-regulation of the alternative exon 8a relative to exon 8 in both cells transfected with siRBM20 and si276-Ex1 (*Figure 6D*). We also observed significant down-regulation of *LMO7* isoforms subject to exons 9 and 10 skipping following knockdown of both siRBM20 and si276-Ex1 (*Figure 6E*). Finally, we detected a significant shift from *CAMK2D*-C, which requires skipping of exons 14–16, to the *CAMK2D*-B isoform, which requires skipping of exon 15, and unspliced *CAMK2D*, in both experimental groups (*Figure 6F* and Supplementary material online, *Figure S12B*). Results were overall strikingly similar between siRBM20 and si276-Ex1, with the only exception of the *CAMK2D*-9 isoform, where knockdown of *RBM20* caused a significant decrease, and knockdown of *TTN-AS1-276* instead caused a significant increase. Next, we confirmed the interaction between RBM20 protein and *CACNA1C*, *LMO7*, and *CAMK2D* RNA by RIP-qPCR (*Figure 6G*). All three genes showed significant enrichment compared to negative control RNA (*GAPDH*) in RBM20-RIP RNA and compared to the signal from non-specific IgG RIP (see Supplementary material online, *Figure S12C*). As expected, the RIP signals for all three genes were almost completely abolished upon *TTN-AS1-276* knockdown (*Figure 6G*). In all, these results support a mechanism where *TTN-AS1-276* is a component of the RBM20 splicing machinery and abets alternative splicing of RBM20 target genes in cardiomyocytes.

### 3.10 TTN-AS1-276 is induced in human heart failure and correlates with TTN I-band exon usage

**Figure 7:**
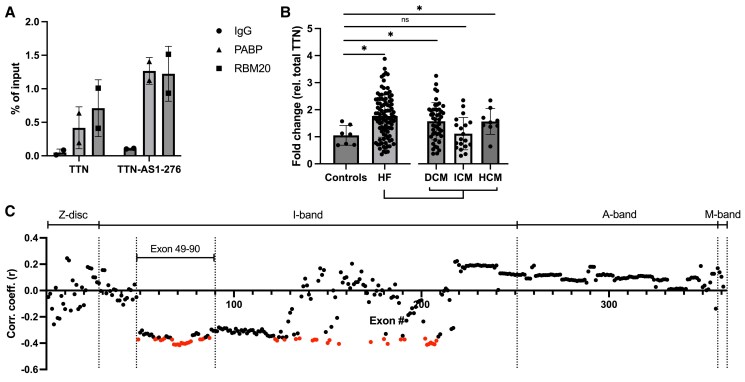
*TTN-AS1-276* expression and correlation with *TTN* splicing in human heart failure. (*A*) RIP was performed on explanted cardiac tissue from two heart failure patients using antibodies against RBM20 or Poly(A)-binding protein (PABP, positive control) or a non-specific IgG antibody (negative control). qRT–PCR was performed to quantify *TTN* and *TTN-AS1-276* in immunoprecipitated RNA. The RIP signal is expressed relative to the 10% input sample. Data points represent the mean of two technical replicates (individual immunoprecipitates) from each patient (*n* = 2). (*B*) Quantification of cardiac *TTN-AS1-276* in a cohort of HF patients (*n* = 100) and unused donor hearts (controls, *n* = 7) using qRT–PCR. Results from the three largest patient subgroups within the HF group, dilated cardiomyopathy (DCM), ischaemic cardiomyopathy (ICM), and hypertrophic cardiomyopathy (HCM) are shown separately to the right. Expression data are normalized to that of total *TTN* and expressed relative to the mean of the controls, *P < 0.05. (*C*) Correlation coefficients from linear regression analyses of *TTN-AS1-276* expression (quantified by qPCR) and PSI for each *TTN* exon in 100 HF patients and seven controls. Statistically significant correlations after Bonferroni correction are shown in red. Exons 49–90, which were among the most affected by *TTN-AS1-276* knockdown in iPS-CM, are also highlighted.

Cardiac *TTN* expression is shifted towards longer and more compliant isoforms in heart failure (HF), but the mechanistic basis for this isoform switch is not known. Given the apparent role of *TTN-AS1-276* in splicing of extensible I-band exons and regulating sarcomere function in iPS-CM, we sought to analyse its expression and function in human HF. First, we confirmed that RBM20 interacts with *TTN-AS1-276* in human heart tissue by performing RBM20 RIP on explanted cardiac biopsies from two HF patients (*Figure 7A*). Then, in order to assess the expression of *TTN-AS1-276* and its association with *TTN* splicing in human HF, we leveraged cardiac RNA-sequencing and qPCR data from a sample of explanted cardiac biopsies from HF patients (*n* = 100) and unused donor hearts (*n* = 7). Patient characteristics are described in *Table 1*. We first assessed the expression of *TTN-AS1-276* with qPCR and observed a marked increase in HF patients compared to controls (*Figure 7B*). Considering the heterogeneity of the HF patient group, we also compared the expression of *TTN-AS1-276* between controls and each of the three major HF aetiologies: dilated cardiomyopathy (DCM, *n* = 47), ischaemic cardiomyopathy (ICM, *n* = 19), and hypertrophic cardiomyopathy (HCM, *n* = 9). Interestingly, increased *TTN-AS1-276* expression was only observed in DCM and HCM. Next, we investigated the association between *TTN-AS1-276* expression and clinical parameters using linear and logistic regression but found no significant correlation between *TTN-AS1-276* and age (*r*^2^ = 0.014; *P* = 0.24) or BMI (*r*^2^ = 0.000032; *P* = 0.99), nor did we observe any significant associations with sex (*β* = −0.13; *P* = 0.7), hypertension (*β* = −0.67; *P* = 0.14), or left ventricular assist device implantation (*β* = −0.16; *P* = 0.76).

To assess whether *TTN-AS1-276* influences splicing of *TTN* I-band exons in human hearts, we used cardiac RNA-sequencing data from the HF cohort to calculate PSI values for each TTN exon (see Supplementary material online, *Figure S13*) and analysed the correlation between PSI and *TTN-AS1-276* expression (*Figure 7C*). After correction for multiple testing, we found a significant negative correlation between *TTN-AS1-276* expression and the PSI of 44 exons, all situated in the I-band. We found no significant positive correlations, nor any significant correlations with exons outside the I-band. These results are in line with our findings *in vitro*, i.e. that *TTN-AS1-276* inhibits inclusion of I-band exons, and provides support for the fact that this mechanism extends to the adult human heart.

## 4. Discussion

In this study, we comprehensively map *TTN* antisense transcription in human heart tissue and define a functional role for the most abundant transcript, *TTN-AS1-276*, in alternative splicing of *TTN*. Based on several lines of evidence from different experimental methods, we postulate that *TTN-AS1-276* interacts with RBM20 to facilitate exon skipping in I-band exons of *TTN*. While it is well established that RBM20 represses exon inclusion in *TTN* and other cardiac genes,^13^ the mechanism by which RBM20 is guided to target pre-mRNA is not well studied. Recently, the splicing factor Srsf1 was shown to be recruited to Triadin (*Trdn*) pre-mRNA via the Triadin antisense transcript (*Trdn-as*) in cardiomyocytes.^19^ Knock out of *Trdn-as* resulted in dysregulated *Trdn* isoform composition, aberrant Ca^2+^-handling and susceptibility to arrhythmias. Our results points to a similar mechanism, where *TTN-AS1-276* facilitates interaction between RBM20 and *TTN* pre-mRNA. While we show that *TTN-AS1-276* co-localizes with both RBM20 and *TTN* mRNA in cardiomyocyte nuclei, that knockdown of *TTN-AS1-276* results in decreased interaction between RBM20 and intronic RBM20-binding motifs in *TTN* and that TTN-AS1-276 seems to interact with RBM20 via its RRM domain, elucidating the structural foundation for this mechanism requires further studies. The RBM20 RRM interacts with intronic UCUU-motifs in target pre-mRNAs^14^ and based on the fact that *TTN-AS1-276* has a comparable density of intronic UCUU-motifs (mean of 9.7/kb across all introns) to introns spanning alternatively spliced exons in the RBM20 target genes studied here, i.e. *TTN* (16/kb), *LMO7* (7.3/kb), *CAMK2D* (9.9/kb), and *CACNA1C* (5.1/kb), we speculate that the interaction with *TTN-AS1* could involve recognition of such motifs by the RBM20 RRM.

Recently, Bertero *et al.*^34^ showed that RBM20 drives genomic reorganization of its target genes through a TID. Here, we show that knockdown of *TTN-AS1-276* affects splicing not only of *TTN*, but several of the other TID-associated RBM20 target genes. The TID includes genes that are involved in cardiomyocyte excitation-contraction coupling (e.g. *CACNA1C* and *CAMK2D*) and it is therefore possible that the effects of *TTN-AS1-276* knockdown on contractility could in part be due to altered Ca^2+^-flux, in addition to the direct effect on TTN isoform composition and sarcomere properties. Future studies will be warranted to study the role of *TTN-AS1-176* in formation and functional consequences of the RBM20 TID beyond TTN splicing. Interestingly, *TTN-AS1* has previously been predicted to be part of other gene regulatory networks in human cardiac tissue. Using bioinformatic tools, Tian *et al.*^35^ showed that *TTN-AS1* was predicted to regulate the Ca^2+^-channel *TRPM5* in the context of right ventricular cardiomyopathy induced by tricuspid regurgitation. Moreover, Zhang *et al.* suggested a potential cardioprotective role for *TTN-AS1* through increasing the stability of *CDK6* mRNA and alleviating hypoxia-induced apoptosis in cardiomyocytes *in vitro*. These results must be taken into account when considering *TTN-AS1* as a potential therapeutic target in heart disease.^36^

We believe that the fact that knockdown of *TTN-AS1-276* in human iPS-CM resulted in a shift towards longer and less stiff sarcomeres with improved diastolic properties merit further investigation into *TTN* antisense transcripts as therapeutic target for diseases characterized by increased myocardial stiffness and diastolic dysfunction, such as HFpEF. Genetic models of RBM20 deficiency have been shown to have improved diastolic function,^37,38^ and we believe that *TTN-AS1* could represent a direct target for regulating RBM20 activity through antisense oligonucleotide (ASO) therapies. The 5′ end of *TTN-AS1-276* does not overlap *TTN* exons (nor those of any other gene) and thus represents a feasible target for specific ASO-mediated knockdown. Moreover, the fact that *TTN-AS1-276* appears to have its own promoter (see Supplementary material online, *Figure S2B*) means that it is amenable to transcriptional modulation by CRISPRi.^39^ Additional studies are required to assess whether interfering with *Ttn* antisense transcription can be harnessed to modulate sarcomere properties and improve cardiac function in *in vivo* models of diastolic dysfunction and HFpEF. One annotated antisense transcript has been identified in the mouse *Ttn* locus (ENSMUST00000156809). As commonly observed among antisense transcripts,^40^ overall sequence conservation with human *TTN* antisense transcripts is low, but it is interesting to note that exon 2 of the mouse transcript has 94% similarity to exon 2 of TTN-AS1-276. An important aspect of therapeutically reducing TTN stiffness has been raised in genetic models of RBM20 loss-of-function. While homozygous deletion of the RBM20 RRM domain caused increased sarcomere length and improved diastolic function, there was a concomitant reduction in maximal systolic stress and a depression of length-dependent activation.^38^ A rigorous evaluation of the therapeutic window and careful dosing of any ASOs targeting *TTN* antisense transcripts will therefore be necessary.

A limitation of the study is the lack of an adult human cardiomyocyte *in vitro* model. iPS-CMs are known to exhibit a foetal phenotype^41^ and have previously been shown to predominantly express longer N2BA isoforms and a considerably higher N2BA:N2B ratio than adult cardiomyocytes.^30^ However, in our live cell imaging experiments, we observe sarcomere length and contraction dynamics that are comparable to adult cardiomyocytes.

In conclusion, we show that antisense transcripts play an integral role in regulation of *TTN* alternative splicing and sarcomere function in cardiomyocytes and could constitute targets for therapeutic modulation of cardiac stiffness.

## Supplementary Material

cvaf037_Supplementary_Data

## Data Availability

All data are available upon reasonable request.
