# Supplementary material for: Antisense-mediated regulation of exon usage in the elastic spring region of Titin modulates sarcomere function
Source: Cardiovasc Res. 2025 Mar 5;121(4):629–42. doi: 10.1093/cvr/cvaf037 (PMC12054628; doi:10.1093/cvr/cvaf037)
Supplement: cvaf037_Supplementary_Data [file cvaf037_supplementary_data.zip › TTN-AS_MS_R1_Supplement.pdf]

Supplemental Material

Antisense-mediated regulation of exon usage in the elastic spring region of Titin modulates sarcomere function

Celik *et al*

Contents:

Supplemental Figures 1-13
Supplemental Tables 1-2
Supplemental Methods

Supplemental Figure 1

a)

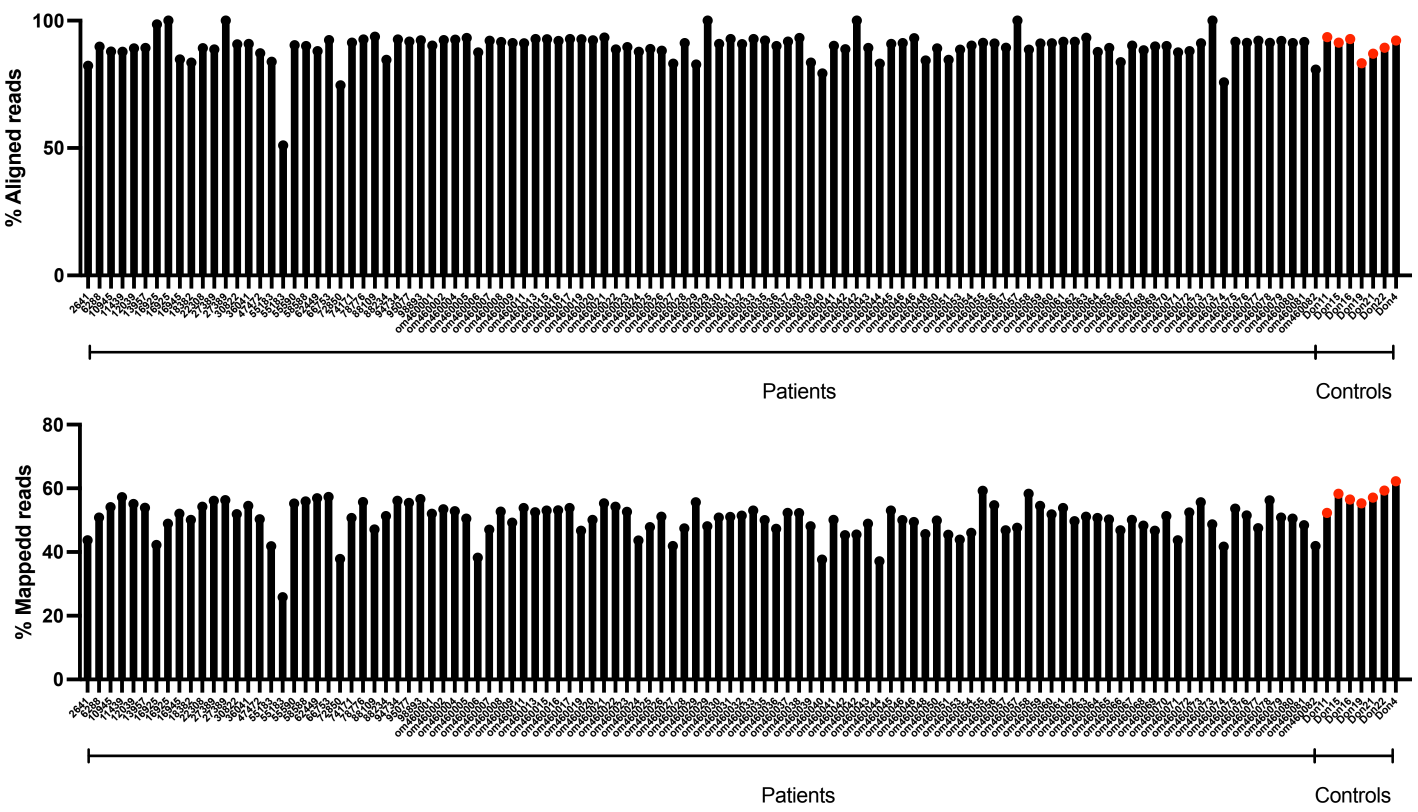

b)

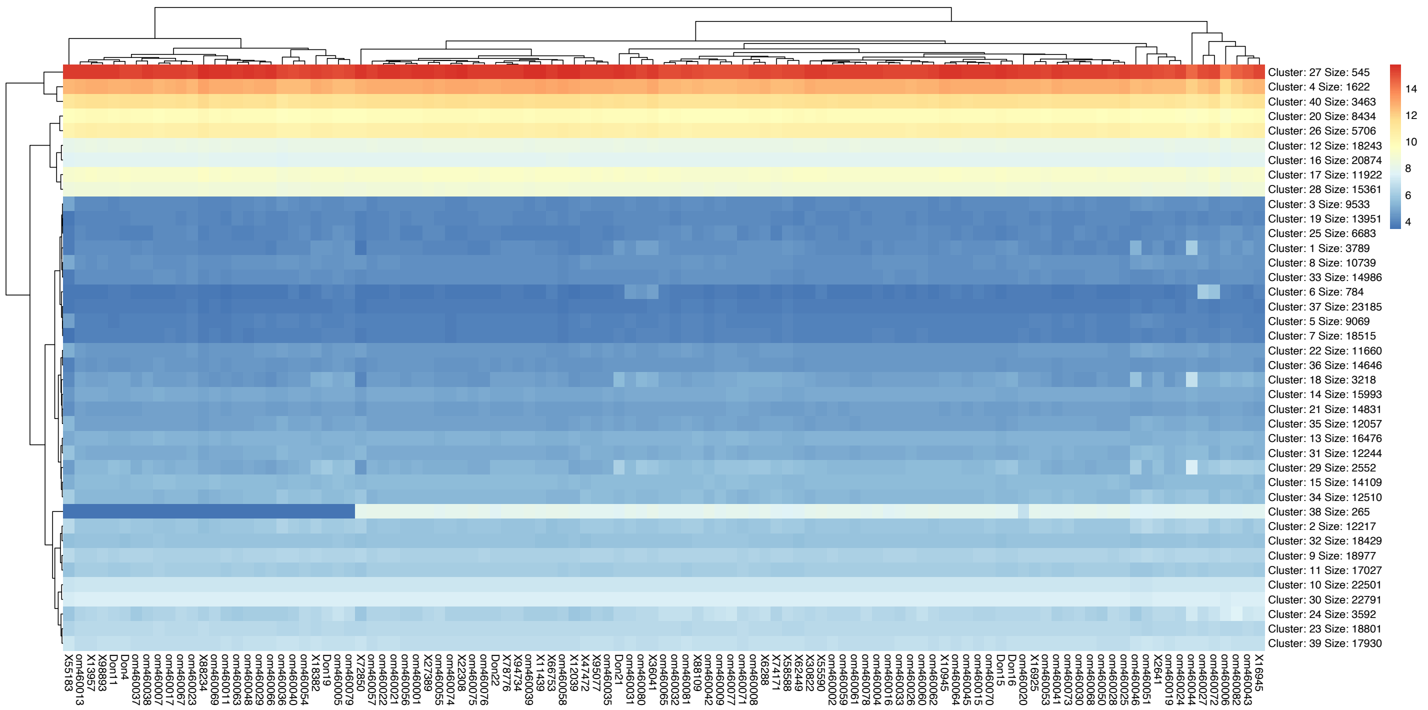

Supplemental Figure 1. Quality control metrics for RNA-Sequencing data. Bulk RNA-sequencing was performed on biopsies from explanted hearts (n=100) and unused organ donor hearts (n=7). A) Proportion of aligned (top) and mapped (bottom) reads in each sample. Unused donor heart samples are indicated by red circles. B) Heatmap showing k-means clustering of normalized exon count data across all samples after filtering out exons with <500 counts. The number of clusters was arbitrarily set to 40.

Supplemental Figure 2

a)

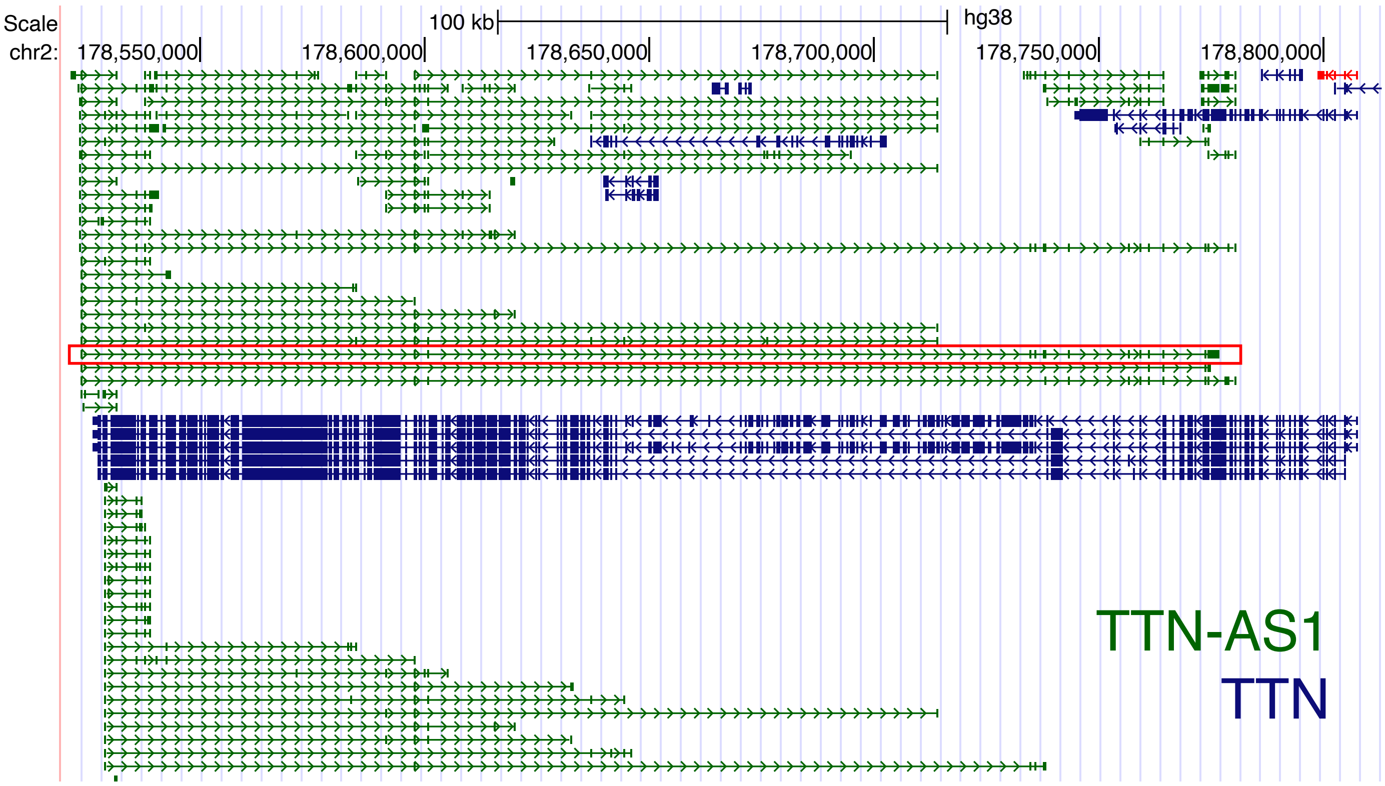

b)

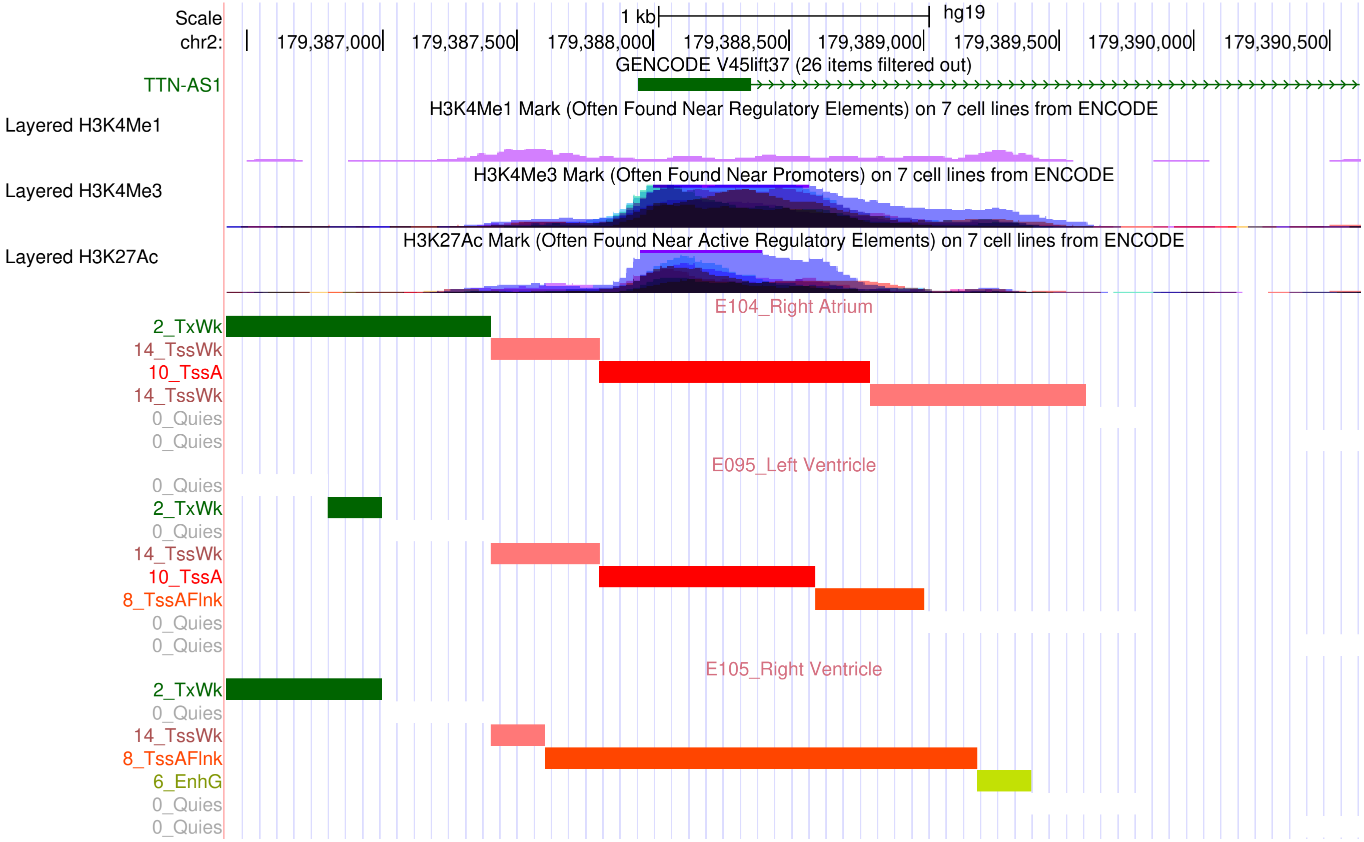

Supplemental Figure 2. Antisense transcription in the *TTN* locus. A) Overview of all annotated GENCODE v. 44 transcripts in the genomic region encompassing *TTN* and *TTN-AS1* genes, as visualized in the UCSC Genome Browser with the GRCh38/hg38 genome assembly. *TTN-AS1* and *TTN* transcripts are colored green and blue, respectively. The transcript highlighted with a red box is the ENSEMBL cannonical transcript, ENST00000659121 or TTN-AS1-276. B) Visualization of epigenomic marks in the TTN-AS1 promoter region. The top track shows Exon 1 of *TTN-AS1-276.* Tracks 2-4 show aggregated ENCODE H3K4Me1, H3K4Me3 and H3K27Ac ChIP-seq read data from 7 cell lines. Tracks 5-7 show functional genomic region annotations based on Integrative and Discriminative Epigenome Annotation System (IDEAS) analysis of Roadmap Epigenomics Consortium data on a core set of histone marks (H3K4me3, H3K4me1, H3K36me3, H3K27me3 and H3K9me3) from cardiac tissues. The red TssA and TssFlnk annotations are indicative of active promoters.

Supplemental Figure 3

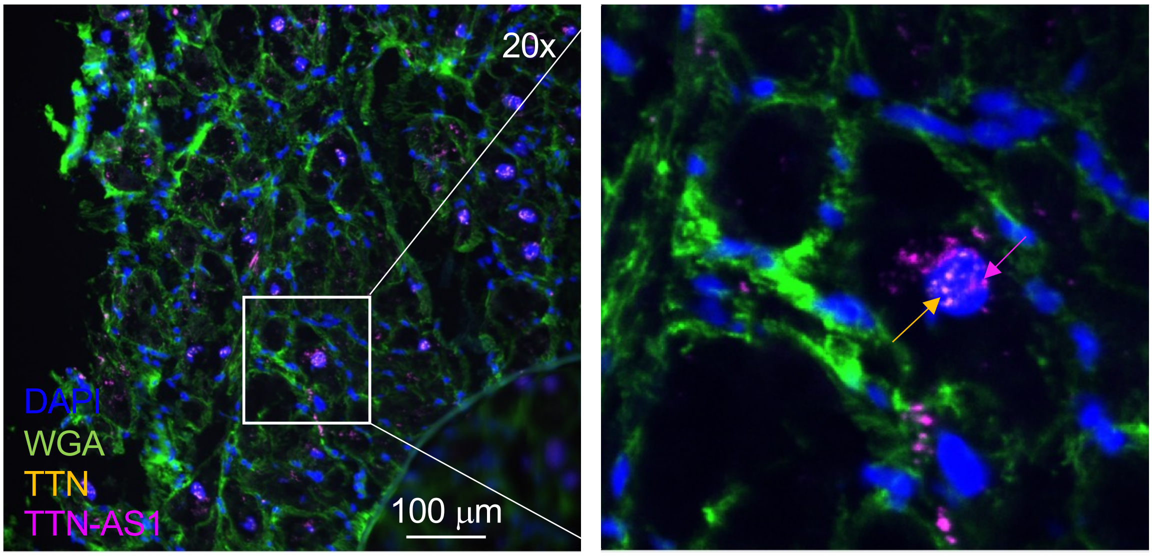

Supplemental Figure 3. Expression and localization of TTN-AS1 in human cardiac tissue. RNA in situ hybridization for *TTN-AS1* (magenta) and *TTN* (yellow) in a tissue section from a human cardiac biopsy. Cell membranes were stained with wheat germ agglutinin (WGA).

Supplemental Figure 4

a)

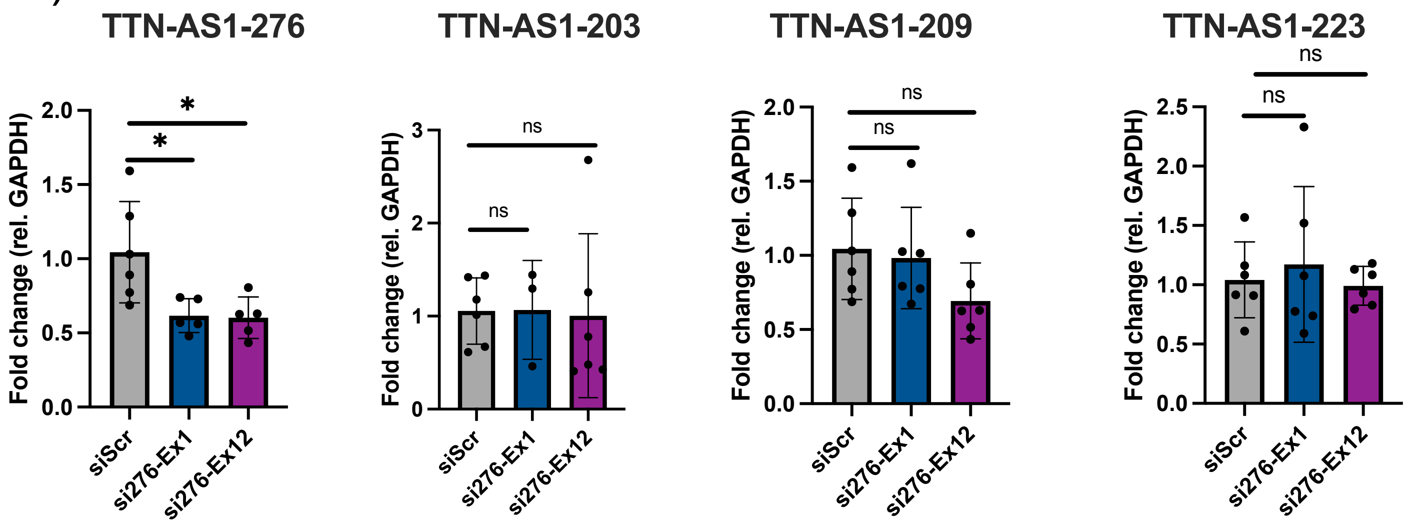

b)

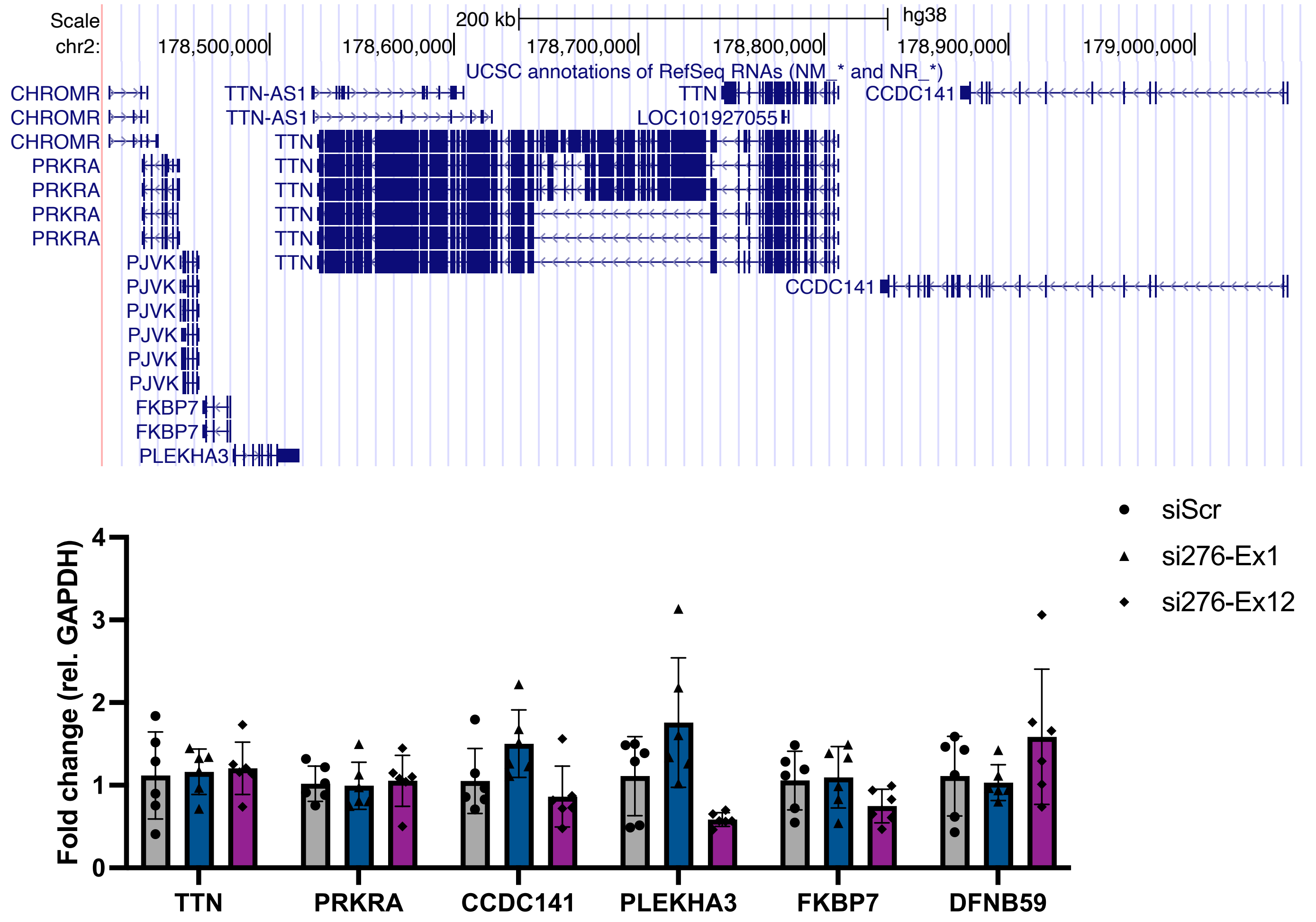

Supplemental Figure 4. Expression of core *TTN-AS1* transcripts and genes in the *TTN* locus following siRNA knock down in iPS-derived cardiomyocytes. Two independent siRNAs were designed to target Exon 1 (si276-Ex1) and 12 (si276-Ex12) of *TTN-AS1-276*, respectively, and transfected into human iCell iPS-derived cardiomyocytes. Expression of (A) *TTN-AS1* transcripts and (B) genes 100 kb up- and downstream of *TTN* (as indicated in the genome browser) were quantified with qRT-PCR assays 48 hours after transfection. *GAPDH* was used as a reference gene for normalization. Data is expressed relative to the negative control group (siScr). The difference in expression between siScr and siTTN-AS1 was assessed with two-sided Student's t-test. n=4-6 per group, *p<0.05.

Supplemental Figure 5

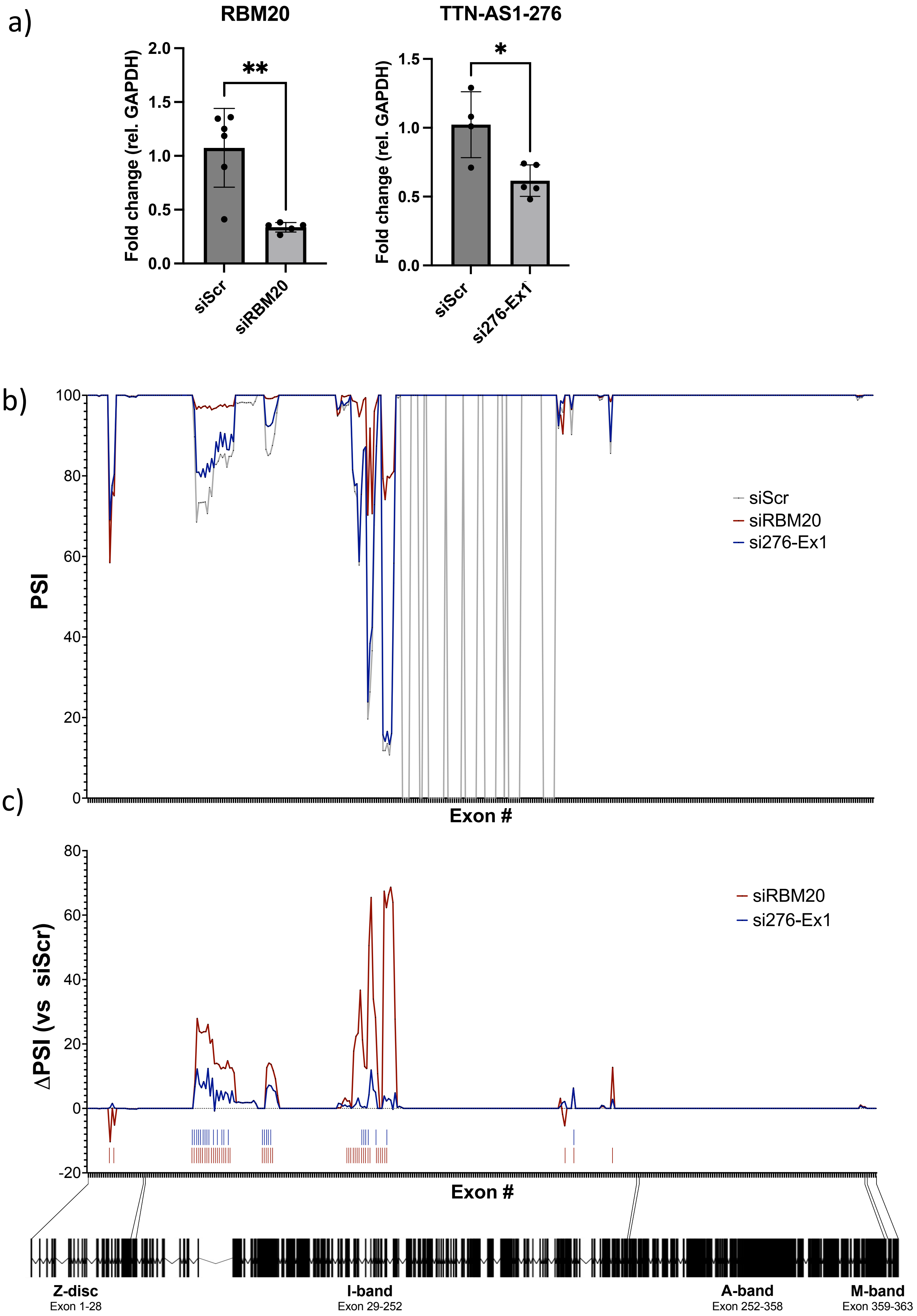

Supplemental Figure 5. Splicing of *TTN* following knock down of *TTN-AS1-276*. A) The expression of *RBM20* and *TTN-AS1-276* was assessed in human iPS-derived cardiomyocytes (iPS-CM) 48 hours after transfection with siRNA towards *RBM20* (siRBM20), *TTN-AS1-276* (si276-Ex1) or scrambled negative control siRNA (siScr) using qRT-PCR. *GAPDH* was used as a reference gene for normalization. Data is expressed relative to the negative control group. The difference in expression between siScr and si276-Ex1 was assessed with two-sided Student's t-test. n=4-6 per group, *p<0.05, **p<0.01. B) Percent spliced in (PSI) for all exons across the *TTN* gene calculated based on RNA-sequencing reads from iPS-CM transfected with siRBM20 (red), si276-Ex1 (blue) or siScr (gray). C) The difference in PSI (ΔPSI) comparing siRBM20 or si276-Ex1 with siScr was assessed using t-tests, adjusting for multiple testing with a false discovery rate of 0.05. Exons with statistically significant differences in PSI are marked with blue (si276-Ex1 vs siScr) and red (siRBM20 vs siScr) bars, respectively.

Supplemental Figure 6

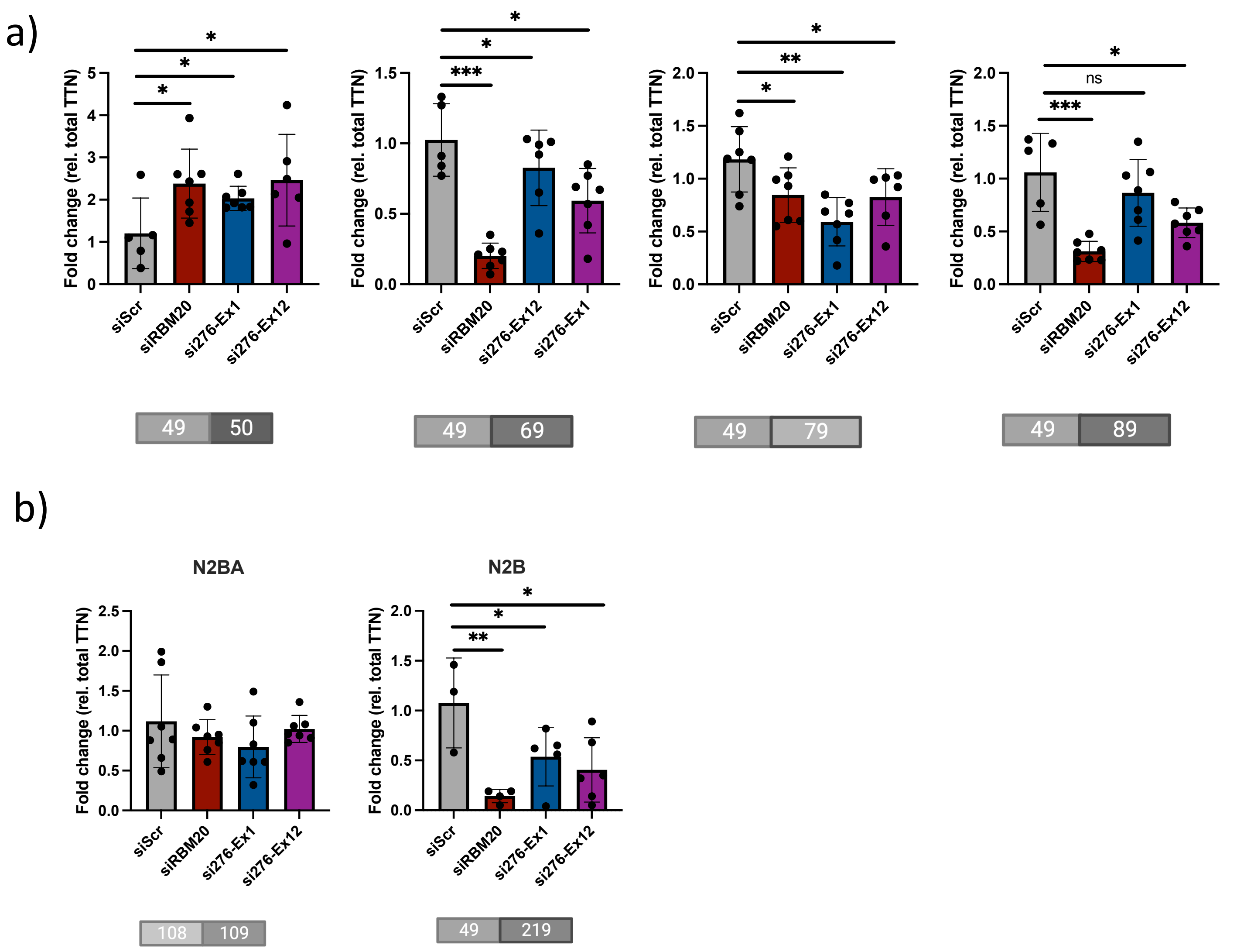

Supplemental Figure 6. Validation of the effect of *TTN-AS1-276* knock down on *TTN* splicing and isoform composition in a separate iPS-CM line. Quantification of *TTN* splice products in human cardiomyocytes derived from the hiPSC line ChIPS22 transfected with si276-Ex1, si276-Ex12, siRBM20 or siScr, using custom qRT-PCR assays spanning the indicated exon-exon junctions. Expression data is normalized to that of total TTN and expressed relative to the mean of the negative control cells (siScr). Data is derived from two separate experiments with 3-6 replicates (individual RNA preparations) per experimental group. Differences between each individual experimental group and the control group were assessed with Student's t-tests, *p<0.05, **p<0.01, ***p<0.001.

Supplemental Figure 7

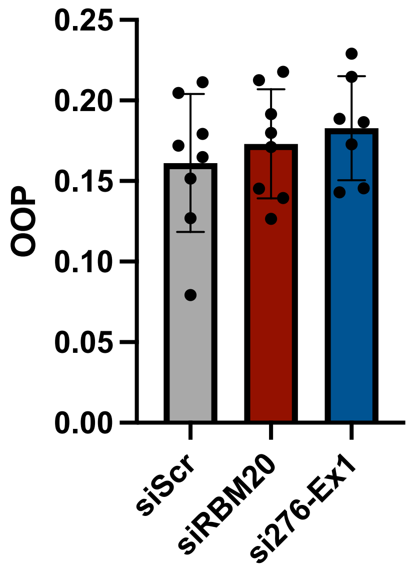

Supplemental Figure 7. Sarcomere organization in iPS-derived cardiomyocytes after knock down of *TTN-AS1*. Live human iPS-derived cardiomyocytes (iPS-CM) expressing GFP-labeled sarcomeric alpha actinin and transfected with siRNA towards *RBM20* (siRBM20), *TTN-AS1-276* (si276-Ex1) or scrambled negative control siRNA (siScr) were filmed during consecutive contractions (n=7-8 replicates). Sarcomere tracking of 14,636 sarcomeres in total was performed in SarcGraph v. 0.2.1 to calculate Orientational Order Parameter (OOP). OOP is a measure of sarcomere organization that ranges from 0 (random orientation) to 1 (perfectly aligned)

Supplemental Figure 8

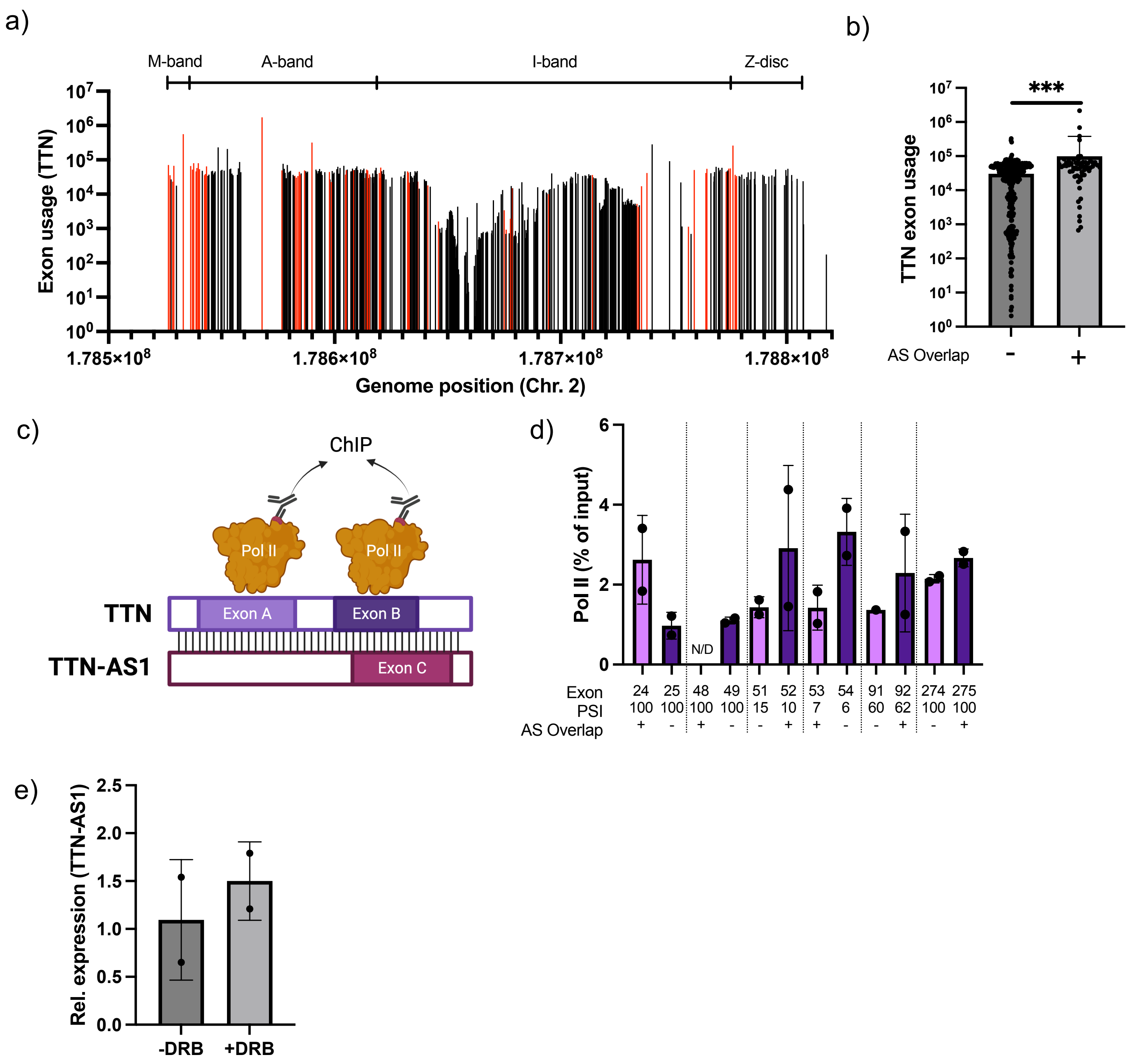

Supplemental Figure 8. Figure 5. No influence of overlapping antisense exons on TTN exon usage. A) Mean TTN exon usage was calculated using cardiac RNA-sequencing data from 7 organ donor hearts without heart disease. Exons with an overlapping TTN-AS1 exon are marked in red. B) Usage of exons with and without an overlapping TTN-AS1 exon. The difference in exon usage between exons with and without an overlapping antisense exon was assessed with Student's t-test, ***p<0.001. C) Depiction of experimental design for PolII ChIP experiments in D). ChIP was performed with a PolII antibody on chromatin from human iPS-derived cardiomyocytes (iPS-CM). PolII occupancy was assessed on six pairs of consecutive TTN exons where one had an overlapping antisense exon and the other did not, using qRT-PCR on ChIP DNA. Data was derived from two separate experiments. E) Relative expression of TTN-AS1 in the chromatin-enriched fraction of iPS-CM nuclei after treatment with DRB, analyzed with qRT-PCR. Data was derived from two separate experiments.

Supplemental Figure 9

a)

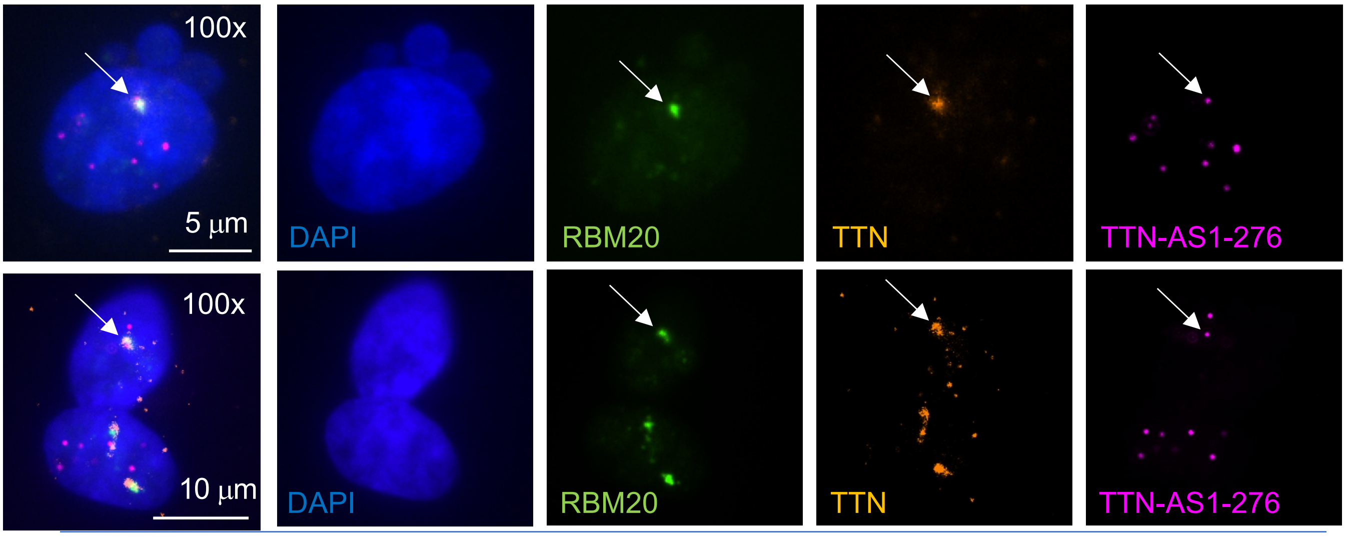

b)

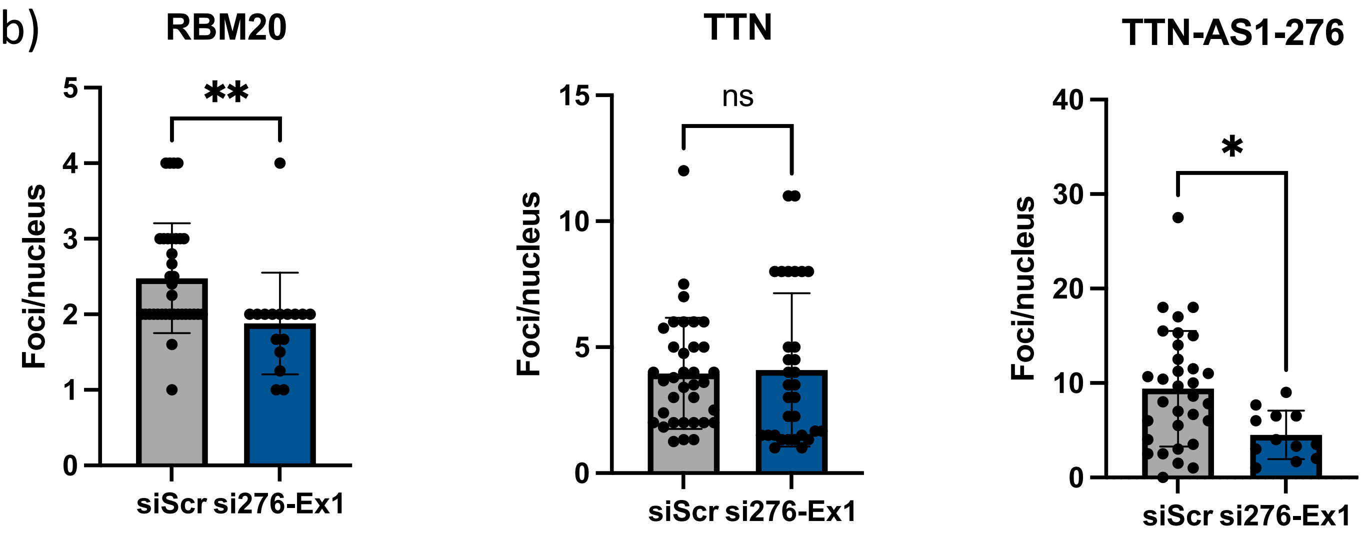

Supplemental Figure 9. Analysis of expression and co-localization of *TTN* and *TTN-AS1* RNA and RBM20 protein in iPS-derived cardiomyocytes. Human iPS-derived cardiomyocytes (iPS-CM) were transfected with siRNA towards *TTN-AS1-276* (si276-Ex1) or scrambled negative control siRNA (siScr) and subjected to combined RNA in situ hybridization for *TTN-AS1-276* (magenta) and *TTN* (orange) and immunofluorescence for RBM20 (green). Nuclei were counterstained with DAPI. A) Representative images of iPS-CM nuclei. White arrows indicate instances of co-localization of *TTN-AS1-276, TTN* and RBM20. B) Quantification of immunofluorescent and in situ hybridization foci, n=16-35 cells per group. Differences between siScr and si276-Ex1 were assessed with two-sided Student's t-test. n=16-35 cells per group, *p<0.05, **p<0.01.

Supplemental Figure 10

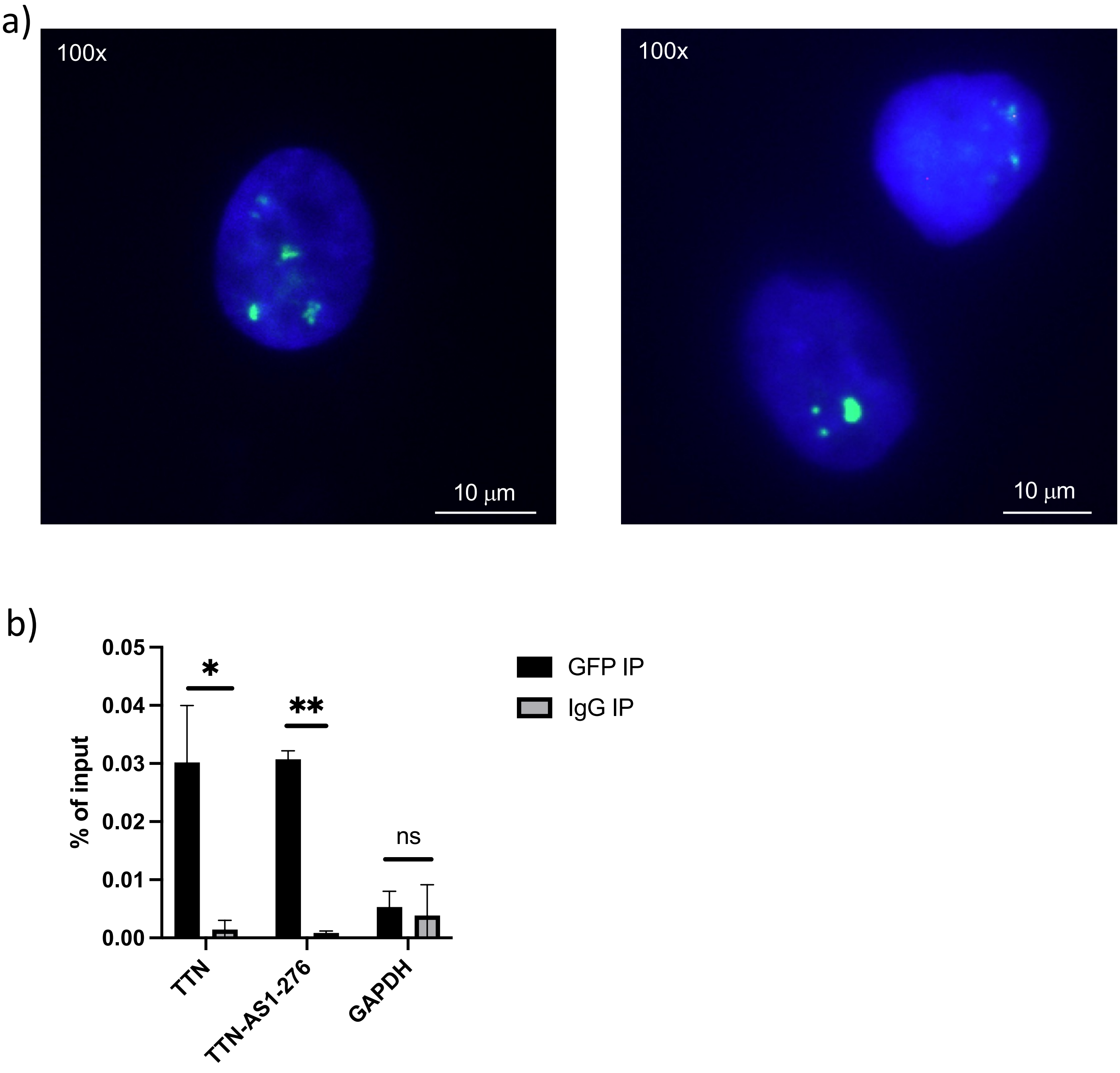

Supplemental Figure 10. Transfection of iPS-derived cardiomyocytes with a RBM20-GFP plasmid for immunoprecipitation. A) Fluorescence imaging of human iPS-derived cardiomyocytes (iPS-CM) after transfection with plasmid expressing an RBM20-GFP fusion protein. Nuclei were counterstained with DAPI. B) Enrichment of *TTN* and *TTN-AS1-276* in GFP-RBM20 RIP RNA compared to negative control IgG IP, analyzed with qRT-PCR. Unrelated *GAPDH* RNA was included as a negative control. Data are derived from two separate experiments with two biological replicates in each group *p<0.05, **p<0.01 using Student's t-tests.

Supplemental Figure 11

a)

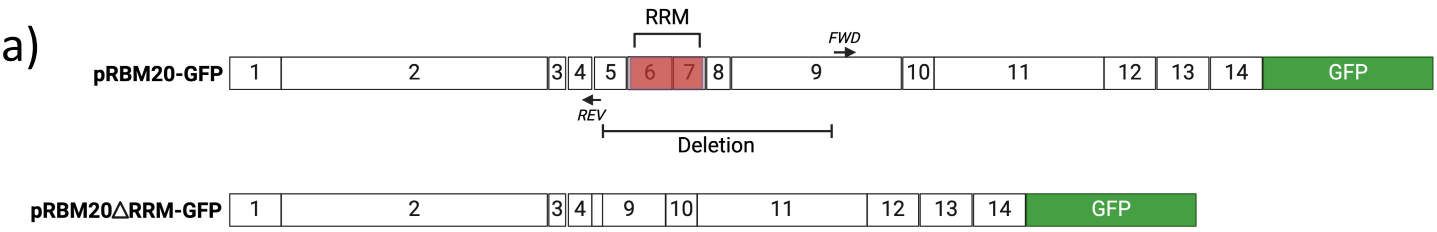

b)

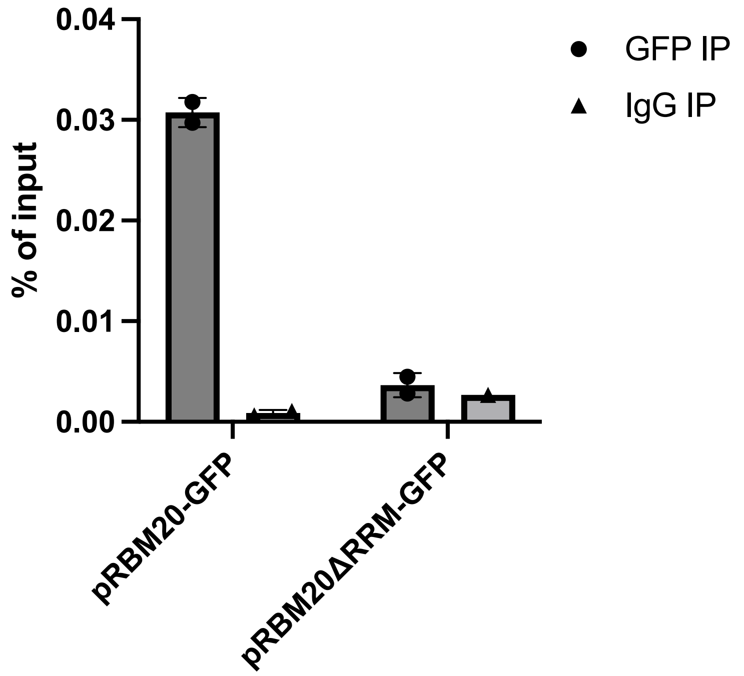

Supplemental Figure 11. Deletion of the RBM20 RRM domain for RNA immunoprecipitation. A) Depiction of the RBM20-GFP open reading frame, indicating the location of the RRM domain and the binding sites for forward (FED) and reverse (REV) primers for site-directed mutagenesis. The lower picture shows the resulting in-frame deletion of exons including the RRM. B) Quantification of *TTN-AS1-276* in GFP RIP RNA from iPS-CM transfected with pRBM20-GFP compared to that from cells transfected with pRBM20∆RRM-GFP, with qRT-PCR. The *TTN-AS1-276* signal from negative control IgG RNA IP are included as negative controls., analyzed with qRT-PCR. Data are derived from two separate experiments with two biological replicates in each group.

Supplemental Figure 12

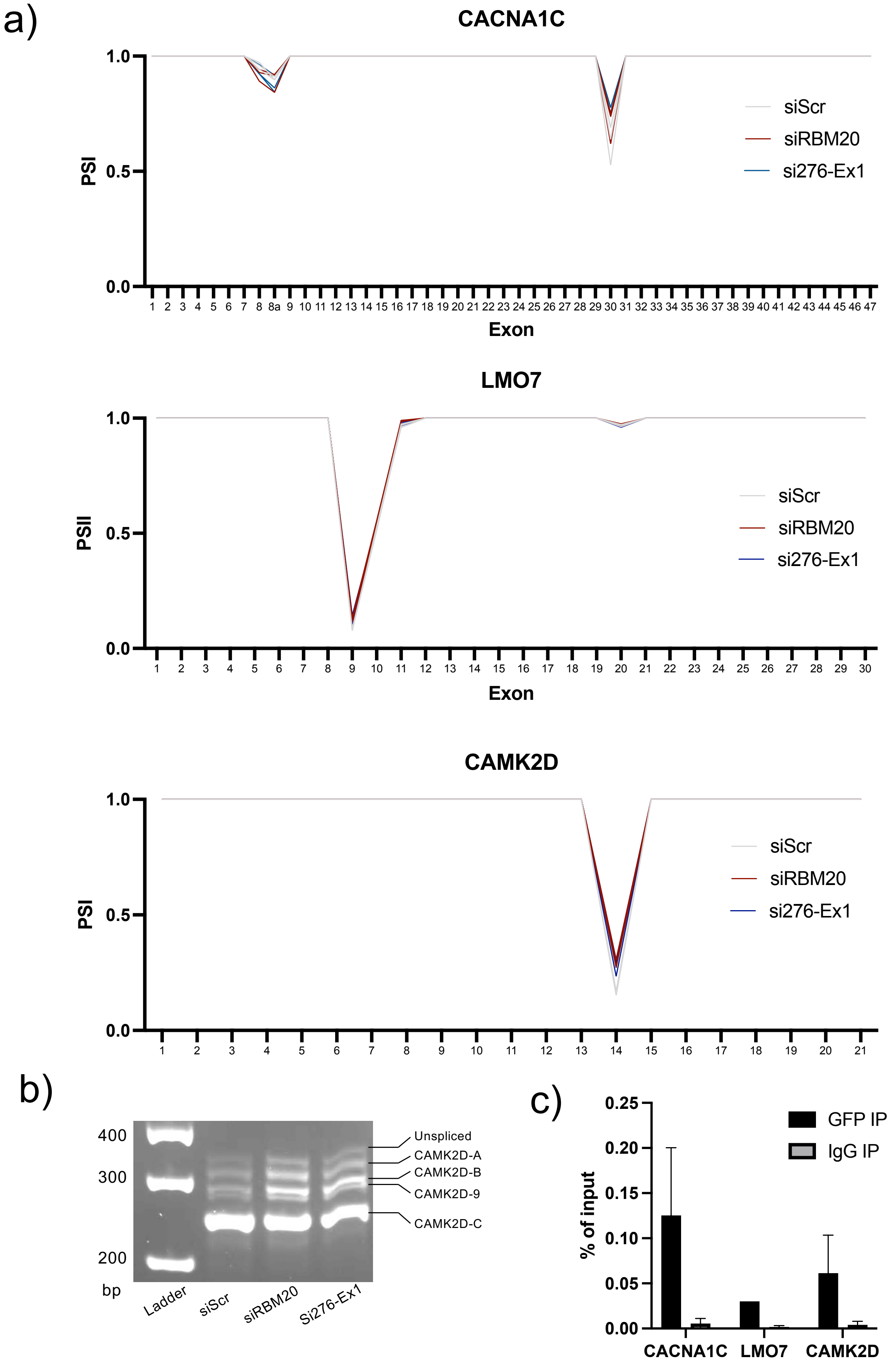

Supplemental Figure 12. Effect of *TTN-AS1-276* knock down on splicing of RBM20 targets. A) Percent spliced in (PSI) for all exons across the *CACNA1C, LMO7 and CAMK2D* genes calculated based on RNA-sequencing reads from human iPS-derived cardiomyocytes (iPS-CM) transfected with siRNA to *RBM20* (red), *TTN-AS1-276* (blue) or scrambled negative control siRNA (siScr, green). B) Semi-quantitative RT-PCR for quantification of *CAMK2D* isoforms. Bands with sizes corresponding to *CAMK2D* isoforms are indicated. C) Enrichment of *CACNA1C*, *LMO7* and *CAMKKD* in GFP-RBM20 RIP RNA compared to negative control IgG IP, analyzed with qRT-PCR. Data are derived from two separate experiments with two biological replicates in each group.

Supplemental Figure 13

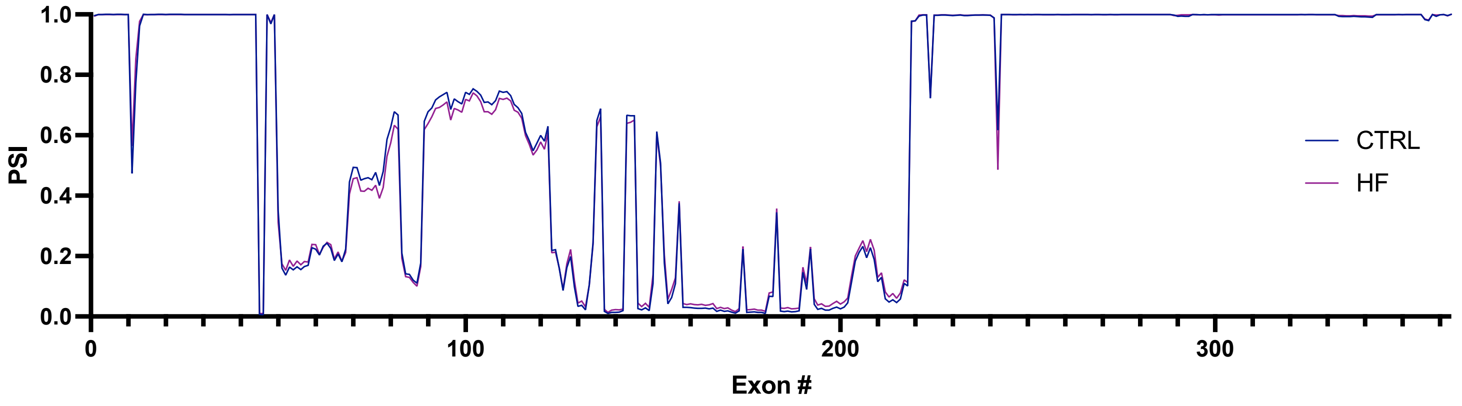

Supplemental Figure 13. Percent spliced in (PSI) across all *TTN* exons in heart failure patients and controls. The mean PSI for each *TTN* exon was calculated based on cardiac bulk RNA-sequencing data from heart failure patients (red, n=100) and unused donor hearts (blue, n=7)

Supplemental Table 1

STROBE Statement - checklist of items that should be included in reports of observational studies

	Item no	Recommendation	Page/Comment
**Title and abstract**	1	a) Indicate the study's design with a commonly used term in the title or the abstract	2
		b) Provide in the abstract an informative and balanced summary of what was done and what was found	2
**Introduction**			
Background/rationale	2	Explain the scientific background and rationale for the investigation being reported	3
Objectives	3	State specific objectives, including any prespecified hypotheses	4
**Methods**			
Study design	4	Present key elements of study design early in the paper	5
Setting	5	Describe the setting, locations, and relevant dates, including periods of recruitment, exposure, follow-up, and data collection	5 + Supplemental Methods
Participants	6	a) Cohort study - Give the elegibility criteria, and the sources and methods of selection of participants. Describe methods of follow-up	5 + Supplemental Methods
		Case-control study - Give the elegibility criteria, and the sources and methods of case ascertainment and control selection. Give the rationale for the choice of cases and controls	
		Cross-sectional study - Give the elegibility critiera, and the sources and methods of selection of participants	
		b) Cohort study - For matched studies, give matching criteria and number of exposed and unexposed	
		Case-control study - For matched studies, give matching criteria and the number of controls per case	

Variables			
	7	Clearly define all outcomes, exposures, predictors, potential confounders, and effect modifiers. Give diagnostic criteria, if applicable	5 + Supplemental Methods
Data sources/management	8*	For each variable of interest, give sources of data and details of methods of assessment (measurement). Describe comparability of assessment methods if there is more than one group	5 + Supplemental Methods
Bias	9	Describe any efforts to address potential sources of bias	5 + Supplemental Methods
Study size	10	Explain how the study size was arrived at	5 + Supplemental Methods
Quantitative variables	11	Explain how quantitative variables were handled in the analyses. If applicable, describe which groupings were chosen and why	10-11 and 32-36
Statistical methods	12	a) Describe all statistical methods, including those used to control for confounding	10-11 and 32-36
		b) Describe any methods used to examine subgroups and interactions	23 and 36
		c) Explain how missing data were addressed	N/A
		d) Cohort study - if applicable, explain how loss to follow-up was addressed	N/A
		Case-control study - If applicable, explain how matching of cases and controls was addressed	
		Cross-sectional study - If applicable, describe analytical methods taking account of sampling strategy	
		e) Describe any sensitivity analyses	N/A
Participants	13*	a) Report numbers of individuals at each stage of study - eg numbers potentially eligible, examined for eligibility, confirmed eligible, included in the study, completing follow-up, and analysed	32-36
		b) Give reasons for non-participations at each stage	N/A
		c) Consider use of flow diagram	
Descriptive data	14*	a) Give characteristics of study participants (eg demographic, clinical, social) and information on exposures and potential confounders	5 and 37
		b) Indicate number of participants with missing data for each variable of interest	N/A

		c) Cohort study - Summarise follow-up time (eg, average and total amount)	N/A	
Outcome data	15*	Cohort study - Report numbers of outcome events or summary measures over time	32-36	
		Case-control study - Report numbers in each exposure category, or summary measures of exposure		
		Cross-sectional study - Report numbers of outcome events or summary measures		
Main results	16	a) Give unadjusted estimates and, if applicable, confounder-adjusted estimates and their precision (eg,, 95% confidence interval). Make clear which confounders were adjusted for and why they were included		23
		b) Report category boundaries when continous variables were categorized	N/A	
		c) If relevant, consider translating estimates of relative risk into absolute risk for a meaningful time period	N/A	
Other analyses	17	Report other analyses done - eg analyses of subgroups and interactions, and sensitivity analyses	N/A	
**Discussion**				
Key results	18	Summarise key results with reference to study objectives	24-26	
Limitations	19	Discuss limitations of the study, taking into account sources of potential bias or imprecision. Discuss both direction and magnitude of potential bias.	24-26	
Interpretation	20	Give a cautious overall interpretation of results considering objectives, limitations, multiplicity of analyses, results from similar studies, and other relevant evidence	24-26	
Generalisability	21	Discuss the generalisability (external validity) of the study results	24-26	
**Other information**				

Funding	22	Give the source of funding and the role of the funders for the present study and, if applicable, for the original study on which the present article is based	27

*Give information separately for cases and controls in case-control studies and, if applicable, for exposed and unexposed groups in cohort a

nd cross-sectionalk studies

Supplemental Table 2. Primers and probes sequences.

Target	Transcript ID	Assay location	Method	Assay type	FWD primer, 5'-3'	REV primer, 5'-3'	Probe, 5'-3'
TTN-AS1-276	ENST00000659121.1	Exon 12	Probe-based qPCR	Custom	GTCCACGATGTCCAGGTAATG	CAGCCCAAAGTCAACTGGTA	/56-FAM/TTGCGGATG/ZEN/AGCTGTCCATTGAGG/3IABkFQ/
TTN-AS1-209	ENST00000456053.5	Exon 5-6 junction	Probe-based qPCR	Custom	TTTACAGTGAGACCAGCAAGG	GATGTCCACGTGGTTCTGATAA	/56-FAM/TTAAGGTTC/ZEN/TCCACAGGCAACCCA/3IABkFQ/
TTN-AS1-223	ENST00000587944.5	Exon 1-2 junction	Probe-based qPCR	Custom	CTTCATGCAATATGCAGAGTGTTC	GAGATGGAAGCTTCGTCTTCTG	/56-FAM/ACTTTCCCT/ZEN/TGTCTCCGTCATCTGTG/3IABkFQ/
TTN-AS1-203	ENST00000419746.5	Exon 6-7 junction	Probe-based qPCR	TaqMan, Hs04962294_m1			
TTN (total)	ENST00000589042.5	Exon 6-7 junction	Probe-based qPCR	TaqMan, Hs00399225_m1			
TTN Exon 49-50	ENST00000589042.5	Exon 49-50 junction	Probe-based qPCR	Custom	GCAAAGCTTCCAATGAGTATGG	TAAGGGACTTAGGTCTGGAGAG	56-FAM/AGGTTGGTG/ZEN/GATATGCCTCTGTCAC/3IABkFQ/
TTN Exon 49-69	ENST00000589042.5	Exon 49-69 junction	Probe-based qPCR	Custom	GCAAAGCTTCCAATGAGTATGG	GAGTCACCTGCTTTCACAATTT	/56-FAM/AGCCACACT/ZEN/AACTGTGACAGAACCAC/3IABkFQ/
TTN Exon 49-79	ENST00000589042.5	Exon 49-79 junction	Probe-based qPCR	Custom	CCAGCCTTGAAATCCTGAGAA	GGAGGTTCTGTCACAGTTAGTG	/56-FAM/TGACTGCGG/ZEN/CGAGTATACATGCAA/3IABkFQ/
TTN Exon 49-89	ENST00000589042.5	Exon 49-89 junction	Probe-based qPCR	Custom	CCAGCCTTGAAATCCTGAGAA	GGGTGGTTCTGTCACAGTTAG	/56-FAM/TGACTGCGG/ZEN/CGAGTATACATGCAA/3IABkFQ/
TTN N2BA	ENST00000591111.5	Exon 108-109 junction	Probe-based qPCR	Custom	CAGAGTTCCAATCCCAACCA	GATAGGAGGAGCAACCACAG	/56-FAM/TCAGAGCAG/ZEN/TGCCACCAGAAGAAA/3IABkFQ/
TTN N2B	ENST00000589042.5	Exon 49-219 junction	Probe-based qPCR	Custom	GCAAAGCTTCCAATGAGTATGG	CTTCTTCCTTTGGTTCAGGTTTAC	/56-FAM/TGTGACAGT/ZEN/GCCTGGAGGTGAAA/3IABkFQ/
GAPDH	ENST00000229239.10	Exon 8	Probe-based qPCR	TaqMan, Hs02786624_g1			
LMO7 (total)	ENST00000377534.8	Exon 27-28 junction	Probe-based qPCR	TaqMan, Hs00245600_m1			
LMO7 Exon 8-9	ENST00000377534.8	Exon 8-9 junction	Probe-based qPCR	Custom	TTTCAAGTAATCAGAGGAGGATTTG	GGTCCAACATTTGGGAAGAAAC	/56-FAM/CATGCCAAA/ZEN/CCCAGGGAATGCTTT/3IABkFQ/
LMO7 Exon 8-11	ENST00000377534.8	Exon 8-11 junction	Probe-based qPCR	Custom	AGCCAGACAAACATGAGGATAA	ACTTACTCCCATTCTCACCATAAA	/56-FAM/ATGGAACAT/ZEN/TTTCAAGACTCTTTCAAA/3IABkFQ/
CACNA1C Exon 8-9	ENST00000399655.6	Exon 8-9 junction	Probe-based qPCR	Custom	GTGCTGTACTGGGTCAATGAT	GCACACCGAGAACCAAGTTA	/56-FAM/AAGGGACTG/ZEN/GCCCTGGATCTATTT/3IABkFQ/
CACNA1C Exon 8a-9	ENST00000399603.6	Exon 8-9 junction	Probe-based qPCR	Custom	ACGTGCTGTACTGGATGC	CACACCGAGAACCAGATTTAGA	/56-FAM/ACGCTATGG/ZEN/GCTATGAGTTACCCTG/3IABkFQ/
RBM20	ENST00000369519.4	Exon 11-12 junction	Probe-based qPCR	TaqMan, Hs1098240_m1			
CAMK2D	ENST00000511664.6	Exon 12-19 junction	RT-PCR	Custom	CAACTATGCTGGCTACAAGGAA	CCCAAAGCTTCAGGTTCAAA	
TTN Exon 24	ENST00000589042.5	Exon 24	RT-PCR	Custom	GCGCATCAAACATGGAGAAAG	GGCTCTAGTGTGGGAATGTAAG	
TTN Exon 25	ENST00000589042.5	Exon 25	RT-PCR	Custom	TCAGTGAGCAGGTCTCCTATAC	CTGGCAGTTTGCCCTTCTAA	
TTN Exon 48	ENST00000589042.5	Exon 48	RT-PCR	Custom	CTGAGACACAGGCAGTTCTATC	GGGCTCTTGGGTGATGTTTA	
TTN Exon 49	ENST00000589042.5	Exon 49	RT-PCR	Custom	CCACCTAGCCAAATTCACCT	GCTGACACTGCCATACTCAT	
TTN Exon 51	ENST00000589042.5	Exon 51	RT-PCR	Custom	AATCAGCACGCCTCCATT	TATCACTGCCGACGTCATTC	
TTN Exon 52	ENST00000589042.5	Exon 52	RT-PCR	Custom	GCAGACATAGTGAGAGGAACAA	CCGACTTCATTAGCAACAACAC	
TTN Exon 53	ENST00000589042.5	Exon 53	RT-PCR	Custom	CACTAGGAGGACAAACCGTTAC	GTTTGGCTTCCAGCTTCATTT	
TTN Exon 54	ENST00000589042.5	Exon 54	RT-PCR	Custom	CAGAACTGATCCAGGTGACAG	CTGCCCACTTCATTGGAAATC	
TTN Exon 91	ENST00000589042.5	Exon 91	RT-PCR	Custom	CACCACCAAGATTTGTGAAGAAG	TGCATCCCAGCATCGTTT	
TTN Exon 92	ENST00000589042.5	Exon 92	RT-PCR	Custom	AAAGTTACCACGGGAGACAC	CAGGGTTCTGCACCTCAAA	
TTN Exon 274	ENST00000589042.5	Exon 274	RT-PCR	Custom	CTTGATGATGGTGGCAGTGA	GCTTTGAAACACATGGTGGAC	
TTN Exon 275	ENST00000589042.5	Exon 275	RT-PCR	Custom	CAGATAAGCCCATTGTGGAAGA	CAGCACATACTCTGAAGACATAGG	
PRKRA	ENST00000325748.9	Exon 4-5 junction	Probe-based qPCR	TaqMan, Hs00269379_m1			
CCDC141	ENST00000443758.7	Exon 19-20 junction	Probe-based qPCR	TaqMan, Hs00892642_m1			
PLEKHA3	ENST00000234453.10	Exon 6-7 junction	Probe-based qPCR	TaqMan, Hs00604551_m1			
FKBP7	ENST00000424785.7	Exon 1	Probe-based qPCR	TaqMan, Hs05043173_s1			
DFNB59	ENST00000644580.2	Exon 4-5 junction	Probe-based qPCR	TaqMan, Hs00419435_m1			

Supplemental Methods

Human samples

Left ventricular biopsies from unused organ donor hearts (n=7) and explanted cardiac tissue from heart failure patients (n=100) were collected at transplantation centers at Lund University Hospital, Lund, Sweden and Sahlgrenska University Hospital, Gothenburg, Sweden, and stored at -80°C. Informed consent was provided by patients or in the case of unused organ donor hearts, a close relative. The study was approved by the Ethics Board at Lund and Gothenburg University, respectively. The study was conducted in concordance with the Declaration of Helsinki. The methodology, conduct and reporting of this study were in accordance with the Strengthening the Reporting of Observational Studies in Epidemiology (STROBE) statement for observational studies. STROBE recommendations for reporting observational studies are available as **Supplemental Table 1**.

Human heart muscle cells

Human iCell iPS-derived cardiomyocytes (iPS-CM) were sourced from FujiFilm Cellular Dynamics Inc. (Madison, WI, USA). Cells were thawed and grown in Plating or Maintenance Medium according to the manufacturer's instructions. All treatments were carried out five days post plating. For validation experiments, iPS-CM derived from the hiPSC line ChiPSC22 were obtained from Takara Bio (Takara Bio Europe, Saint-Germain-en-Laye, France). Cryopreserved cells were thawed in Advanced RPMI 1640 medium (ThermoFisher), supplemented with 1x B27 (ThermoFisher), 1x Glutamax (ThermoFisher) and Y27632 (10 μM, StemCell Technologies, Vancouver, Canada) and 20% Fetal Bovine Serum (FBS, ThermoFisher) and seeded in cell culture plates coated with 50 mg/ml Fibronectin

(ThermoFisher). One day after thawing, cell medium was changed to Advanced RPMI 1640 medium supplemented with 1x B27 and 1x Glutamax. Experiments were initiated 7 days post-thaw.

RNA isolation

25 mg of frozen tissue (see above) was cut into small pieces using a scalpel and transferred to a 2 ml tube. Tissue was homogenized in 700 μl of QIAzol (Qiagen, Hilden, Germany) using an Omni TH rotor-stator homogenizer equipped with disposable Hard Tissue Probes. For isolation of RNA from cells, 700 μl of QIAzol was added directly to cell culture plates after removal of culture medium and washing once with PBS. For isolation of chromatin-enriched and soluble nuclear RNA fractions from cells, the protocol described by Werner et al[1] was used. Total RNA was isolated using the miRNeasy mini kit (Qiagen) according to the manufacturer's instructions. The quantity and quality of isolated RNA was assessed with Qubit Flex (ThermoFisher) using the QuantIT RNA HS Assay Kit (ThermoFisher) and Agilent 4200 TapeStation (Agilent Technologies, Santa Clara, CA, USA) using the RNA ScreenTape Analysis Kit (Agilent Technologies).

siRNA and plasmid DNA transfection

For knock down experiments, cells were transfected with Silencer Select siRNA (ThermoFisher) directed towards exon 1 (si276-Ex1, #n294437) or exon 12 (si276-Ex12, custom design ID #ABRSBMG) of TTN-AS1-276, towards *RBM20* (ENST00000369519.4, #s49081) or with a scrambled negative control siRNA sequence (#4390843). For visualization and tracking of sarcomeres, cells were transfected with a plasmid expressing *ACTN2* (*NM_001103.4*) with a eGFP tag.[2] The plasmid was a gift from Johannes Hell (Addgene

plasmid ref #52669). For immunoprecipitation of RBM20, cells were transfected with a pEZ-M03 vector (GeneCopoeia, Rockville, MD, USA) expressing RBM20 (ENST00000369519.4) with an eGFP tag. An in-frame deletion of a region spanning from exon 5 to exon 9 of the RBM20 open reading frame was made using site-directed mutagenesis (SDM) in order to produce a fusion protein lacking the RBM20 RNA recognition motif (RRM). SDM was carried out using the Phusion Site-Directed Mutagenesis Kit (ThermoFisher) according to the manufacturer's instructions with forward primer 5'-GAGCCCAAAGCCAAGTCGGACAAGTAT-3' and reverse primer 5'-CCTTGCTGGAATGGGCACGTATGATGTT-3'. Confirmation of the deletion was performed with PCR using the forward primer 5'-ATAACCCTGCTGGGAATGAAG-3' and reverse primer 5'-CCACTGATTGAGGGCTTTCT-3'. Transfections were performed using Lipofectamine 3000 (ThermoFisher) according to the manufacturer's instructions.

RNA in situ hybridization of human cardiac tissue

10 μm human cardiac cryosections from frozen tissue biopsies (see above) were fixed, dehydrated and pre-treated with Protease IV for the RNAScope Fluorescent Multiplex Assay (Advanced Cell Diagnostics, Hayward, CA, USA) according to the manufacturer's recommendations (ACD User Manual "320513-USM"). The RNAScope Multiplex Fluorescent Assay was performed using the probe Hs-TTN-C1 (#550361, Advanced Cellular Diagnostics) targeting the ubiquitously expressed exons 24-28 in *TTN* (ENST00000456053.5) and the probe Hs-TTN-AS1-C2 (#1115141-C2, Advanced Cellular Diagnostics) targeting exons 1-5 in *TTN-AS1* (ENST00000456053.5) using the Amp4 Alt C-FL option, according to the manufacturer's recommendations (ACD User Manual "320293-USM"). Before mounting, 10 μg/ml Wheat Germ Agglutinin-AlexaFluor488 (ThermoFisher) was added to the sections for 10 minutes. Sections were then washed 3x5 min in PBS, counterstained with DAPI for 30 s

and mounted using ProLong Gold Antifade Mountant. Sections were imaged using a Nikon TiE TIRF microscope (Nikon Corporation, Tokyo, Japan) equipped with a Photometrics Prime95B sCMOS camera (Teledyne Photometrics, Tucson, AZ, USA).

Combined RNA in situ hybridization and immunofluorescence

50,000 human iPS-CM were seeded/well in Lab-Tek 4-well chamber slides (Sigma-Aldrich) and cultured for five days according to the manufacturer's instructions. Cells were then fixed, de- and rehydrated and treated with Protease III in preparation for RNAScope Multiplex Fluorescent Assay (Advanced Cellular Diagnostics) according to the manufacturer's instructions (ACD Technical Note "320538"). The RNAScope Multiplex Fluorescent Assay was performed according to the manufacturer's recommendations (ACD User Manual "320293-USM") with the probes mentioned above. Before mounting, immunofluorescent staining was performed according to the ACD Technical Note "323100-TN". Slides were blocked with 3% BSA in TBS and stained with a rabbit anti-RBM20 antibody (Abcam, #ab233147) at 10 µg/ml for 1 hour. An AlexaFluor488 anti-rabbit IgG secondary antibody (#4412 Cell Signaling, Danvers, MA, USA) at 1:1000 dilution was then added and incubated for 30 minutes. Slides were counterstained with DAPI for 30 s and mounted using ProLong Gold Antifade Mountant. Slides were imaged using Operetta CLS high content screening instrument (PerkinElmer, Waltham, MA, USA) and the number and localization of *TTN* (Atto 550), *TTN-AS1* (Atto 647) and RBM20 (AlexaFluor488) fluorescent foci were analysed using Harmony 5.2 software.

Protein gel electrophoresis

Analysis of TTN protein isoforms was performed with agarose gel electrophoresis according to a previously established protocol.[3] Briefly, human iPS-CM (200,000/well) were seeded in 6-well plates, cultured and transfected with siRNA as described elsewhere. At the end of the experiment, cells were washed once with PBS, scraped in 100 μl of sample buffer (8 M Urea, 2 M Thiourea, 0.05 M Tris-HCl, 75 mM DTT and 3% SDS) and transferred to tubes. Mouse cardiac tissue was frozen in liquid nitrogen, pulverized in a cryogrinder and homogenized in sample buffer using a dounce homogenizer. All samples were heated at 60 °C for 10 minutes, cleared by centrifugation at 13,000x*g* for 5 minutes and stored at -80 °C . A 1.5 mm thick 16x16 cm 1% SeaKem Gold agarose (Lonza group ltd., Basel, Switzerland) gel was cast and used for electrophoresis. 0.5 % 2,2,2-Trichloroethanol (Sigma-Aldrich) was added to the gel to allow for stain-free visualization of proteins. Protein samples were mixed with 4x Laemmli sample buffer supplemented with 0.1 volumes of β-mercapto ethanol before running the gel. ~20 μl of sample was added per well and electrophoresis was allowed to run at 60V until the dye front ran out of the stacking gel (~5-6 h). Protein bands were visualized on ChemiDoc MP imaging system (Bio-Rad) using "Stain-free gel" settings. Bands representing TTN isoforms were quantified using densiometric measurements in Image Lab 6.1 (Bio-Rad) and normalized to the myosin heavy chain 7 (MYH7) band.

RNA Immunoprecipitation

Human iPS-CM were seeded in 60 mm cell culture dishes (800,000 cells/dish) and cultured according to the manufacturer's instructions. After five days, cells were transfected with pCMV-RBM20-GFP plasmid DNA and siRNA as described elsewhere. 72 hours after transfection, cells were washed twice, scraped in 1 ml of ice-cold PBS and pelleted by centrifugation. 200 μl of mild lysis buffer (Merck) was added and incubated on ice for 15

minutes. The cell lysate was cleared by centrifugation at 16,000x*g* for 10 minutes. RNA immunoprecipitation was performed on 100 µl cell lysate per sample using the Magna RIP Kit (Merck) according to the manufacturer's instructions. 5 µg of rabbit polyclonal anti-GFP antibody (#ab290, Abcam) or 1 µg of rabbit IgG antibody was used per immunoprecipitation. For human cardiac samples, ~800 mg pieces of tissue were placed in a petri dish on ice, washed three times with PBS, minced with a scalpel and homogenized in 1 ml of ice-cold PBS in a Dounce homogenizer with 15-20 strokes. Cells were collected by centrifugation at 1500 rpm for 5 minutes at 4 °C and resuspended in 200 µl of RIP Lysis buffer. RIP was performed using the Magna RIP Kit (Merck) according to the manufacturer's instructions with 5 µg rabbit anti-RBM20 antibody (Abcam, #ab233147), 5 µg PABP antibody (#PA5-17599, ThermoFisher) or 1 µg of rabbit IgG antibody

Chromatin immunoprecipitation

Human iPS-CM were seeded in 10 cm cell culture dishes ($2x10^6$ cells/dish) cultured according to the manufacturer's instructions. Preparation of cross-linked chromatin and chromatin immunoprecipitation was performed using the SimpleChIP Kit (CellSignaling) according to the manufacturer's instructions. 5 µg of crosslinked chromatin and 1 µg of ChIPAb+ anti-RNA Pol II mouse monoclonal antibody or mouse IgG antibody was used per immunoprecipitation.

Sarcomere tracking

iPS-CM (~30,000) were seeded in the middle lower right chamber (Pattern 11) of a U-Slide 8 well^{high} u-Pattern^{RGD} chamber slide (Ibidi GmbH, Grafelfing, Germany). After five days in culture, cells were transfected with siRNA to knock down RBM20 or TTN-AS1-276, and

pACTN2-GFP to visualize the sarcomere Z-discs. The procedure for transfection is described in detail above. 48h after transfection, live cell imaging of fluorescently labeled, contracting iPS-CM was performed with wide-field epifluorescence microscopy using a ECLIPSE Ti2-E microscope (Nikon) equipped with a SPECTRA X light engine (Lumencor Inc, Beaverton, OR, USA) and a 60X/1.27 numerical aperture CFI SR Plan Apo objective. Videos of contracting cells, capturing at least two contractions (~2-3 seconds), were recorded at 30 frames per second using a Nikon DS-Qi2 CMOS camera. Segmentation of z-discs and sarcomere tracking was then performed on a total of 32 video files in the SarcGraph Software.[4]

qPCR and RT-PCR

cDNA was synthesized using the RevertAid FirstStrand cDNA Synthesis Kit with random hexamer primers (ThermoFisher) according to the manufacturer's instructions and used in qPCR reactions with 2x Universal TaqMan Master Mix (ThermoFisher) or in RT-PCR reactions with 2x PCR Master Mix (ThermoFisher). The expression of *TTN*, *RBM20*, *GAPDH, CAMK2D, CACNA1C, LMO7, PRKRA, CCDC141, PLEKHA3, FKBP7* and *DFNB59* was assessed with TaqMan Gene Expression Assays (ThermoFisher). For the quantification of specific splice products or exons from *TTN*, *TTN-AS1*, *CAMK2D*, *CACNA1C* and *LMO7*, custom PrimeTime qPCR Probe Assays spanning specific exon-exon junctions or within exons were designed using the PrimerQuest Tool (Integrated DNA Technologies, Coralville, IA, USA). See **Supplemental Table 2** for primer and probe sequences. All qPCR reactions were run on a Bio-Rad CFX 96 instrument (Bio-Rad, Hercules, CA, USA). For gene expression analysis, Ct-values were normalized to the reference gene *GAPDH* and expressed relative to the mean of the control group. For quantification of splicing products/isoforms, Ct-values were normalized to a qPCR assay designed to measure all transcripts from the corresponding gene and expressed

relative to the mean of the control group. For quantification of immunoprecipitated RNA, Ct-values were transformed to ”% of input” using the Ct-value of the 2% Input sample with the formula 2^-(Ct_{RIP}-(Ct_{Input}-3,32). RT-PCR reactions were run on a 2% agarose gel with GelRed and amplicons were visualized using a ChemiDoc MP imaging system with the "UV Trans" application. Bands corresponding to the PCR amplicons were quantified using densiometric measurements in Image Lab 6.1 and normalized to the input samples.

RNA-sequencing

For human cardiac tissue samples, 800 ng of RNA was used as input for library preparation using the TruSeq Stranded Total RNA Library Prep Gold kit (Illumina, San Diego, CA, USA) with rRNA Removal Mix. Sequencing was performed on a NovaSeq 6000 system using the NovaSeq 6000 S4 reagent kit (Illumina) with 101 bp paired-end reads.

Raw fastq files were processed using the nf-core RNAseq/3.12.0 pipeline.[5, 6] Trimming of adapter sequences and removal of low-quality reads were done using Trim Galore 0.6.7[7] and rRNA was removed by SortMeRNA 4.3.4[8] with rRNA database smr_v4.3_default_db.fasta. The quality of the fastq files generated by SortMeRNA 4.3.4 was evaluated using FastQC 0.11.9. BBmap 38.61b[9] was used to map the clean reads to the human reference genome (Homo_sapiens.GRCh38.dna_sm.primary_assembly.fa.gz). Some samples were processed using the BBmap function repair.sh before being fed to BBmap for mapping. The generated mapped bam files were sorted and indexed with samtools 1.17.[10] Mapping quality was evaluated with samtools 1.17[11] and the results were aggregated together with the abovementioned FastQC results using MultiQC 1.12 (**Supplemental Figure 1a**). The biotype content of the reads was counted with the featureCount program from the subread 2.0.3 package.[12] The unique mapping rate was also estimated by counting the reads with NH:i:1

using samtools 1.17. The mapped reads were then counted with DEXSeq 1.44.0 using only reads with a mapping quality>10. Counting used the feature annotation from *Homo_sapiens.GRCh38.109.gtf.gz*. The DEXSeq script dexseq_prepare_annotation.py was used to collapse exon information to define exon counting bins. Exon bin counts were processed and quantified using DexSeq. As an additional quality control measure, k-means clustering was performed on normalized exon count data after removal of exon bins with read count<500 and variance stabilizing transformation (VST). The number of clusters were arbitrarily set to 40 (**Supplemental Figure 1b**). Percent spliced in (PSI) for each exon of the *TTN* gene was calculated with the Calculate-PSI package (https://github.com/jalwillcox/Calculate-PSI).

For human iPS-derived cardiomyocytes, 10 ng of RNA was used as input for cDNA synthesis using the SMART-Seq v4 Ultra Low Input RNA Kit for Sequencing (Takara Bio, San Jose, CA, USA). Library preparation was performed using the Nextera XT DNA Library Preparation Kit (Illumina). Sequencing was performed using the NovaSeq 6000 SP Reagent Kit v 1.0 (Illumina) with 101 bp paired-end reads.

Demultiplexing was performed using the bcl2fastq2 software (RRID:SCR_015058) with default settings. Reads were aligned to the human GRCh38 reference genome with the gencode version 33 annotation, using the STAR software.[13] Quantification of gene expression was performed using the featureCounts software. Raw gene counts were transformed to fragments per kilobase of exon per million mapped fragments (FPKM) using an in-house python script, fCounts2fpkm.py.

All mapped reads were then counted with DEXseq 1.44.0[14, 15] following the instructions at https://rdrr.io/bioc/DEXSeq/f/vignettes/DEXSeq.Rmd. Only reads with mapping quality ≥10 were counted. The counting used the feature annotation information from

Homo_sapiens.GRCh38.109.gtf.gz

(https://www.gencodegenes.org/human/release_33.html). Percent spliced in (PSI) was estimated using the Calculate-PSI package (https://github.com/jalwillcox/Calculate-PSI).

References

1. Werner MS and Ruthenburg AJ. Nuclear Fractionation Reveals Thousands of Chromatin-Tethered Noncoding RNAs Adjacent to Active Genes. *Cell Rep*. 2015;12:1089-98.
2. Hall DD, Dai S, Tseng PY, Malik Z, Nguyen M, Matt L, Schnizler K, Shephard A, Mohapatra DP, Tsuruta F, Dolmetsch RE, Christel CJ, Lee A, Burette A, Weinberg RJ and Hell JW. Competition between alpha-actinin and Ca(2)(+)-calmodulin controls surface retention of the L-type Ca(2)(+) channel Ca(V)1.2. *Neuron*. 2013;78:483-97.
3. Greaser ML and Warren CM. Method for resolution and western blotting of very large proteins using agarose electrophoresis. *Methods Mol Biol*. 2015;1312:285-91.
4. Zhao B, Zhang K, Chen CS and Lejeune E. Sarc-Graph: Automated segmentation, tracking, and analysis of sarcomeres in hiPSC-derived cardiomyocytes. *PLoS Comput Biol*. 2021;17:e1009443.
5. Ewels PA, Peltzer A, Fillinger S, Patel H, Alneberg J, Wilm A, Garcia MU, Di Tommaso P and Nahnsen S. The nf-core framework for community-curated bioinformatics pipelines. *Nat Biotechnol*. 2020;38:276-278.
6. Di Tommaso P, Chatzou M, Floden EW, Barja PP, Palumbo E and Notredame C. Nextflow enables reproducible computational workflows. *Nat Biotechnol*. 2017;35:316-319.
7. Kovaka S, Zimin AV, Pertea GM, Razaghi R, Salzberg SL and Pertea M. Transcriptome assembly from long-read RNA-seq alignments with StringTie2. *Genome Biol*. 2019;20:278.
8. Kopylova E, Noe L and Touzet H. SortMeRNA: fast and accurate filtering of ribosomal RNAs in metatranscriptomic data. *Bioinformatics*. 2012;28:3211-7.
9. Quinlan AR and Hall IM. BEDTools: a flexible suite of utilities for comparing genomic features. *Bioinformatics*. 2010;26:841-2.
10. Li H, Handsaker B, Wysoker A, Fennell T, Ruan J, Homer N, Marth G, Abecasis G, Durbin R and Genome Project Data Processing S. The Sequence Alignment/Map format and SAMtools. *Bioinformatics*. 2009;25:2078-9.
11. Wang L, Wang S and Li W. RSeQC: quality control of RNA-seq experiments. *Bioinformatics*. 2012;28:2184-5.
12. Liao Y, Smyth GK and Shi W. The Subread aligner: fast, accurate and scalable read mapping by seed-and-vote. *Nucleic Acids Res*. 2013;41:e108.
13. Dobin A, Davis CA, Schlesinger F, Drenkow J, Zaleski C, Jha S, Batut P, Chaisson M and Gingeras TR. STAR: ultrafast universal RNA-seq aligner. *Bioinformatics*. 2013;29:15-21.
14. Anders S, Reyes A and Huber W. Detecting differential usage of exons from RNA-seq data. *Genome Res*. 2012;22:2008-17.
15. Reyes A, Anders S, Weatheritt RJ, Gibson TJ, Steinmetz LM and Huber W. Drift and conservation of differential exon usage across tissues in primate species. *Proc Natl Acad Sci U S A*. 2013;110:15377-82.
