# Supplementary figures and images for: Antisense-mediated regulation of exon usage in the elastic spring region of Titin modulates sarcomere function
Source: Cardiovasc Res. 2025 Mar 5;121(4):629–42. doi: 10.1093/cvr/cvaf037 (PMC12054628; doi:10.1093/cvr/cvaf037)

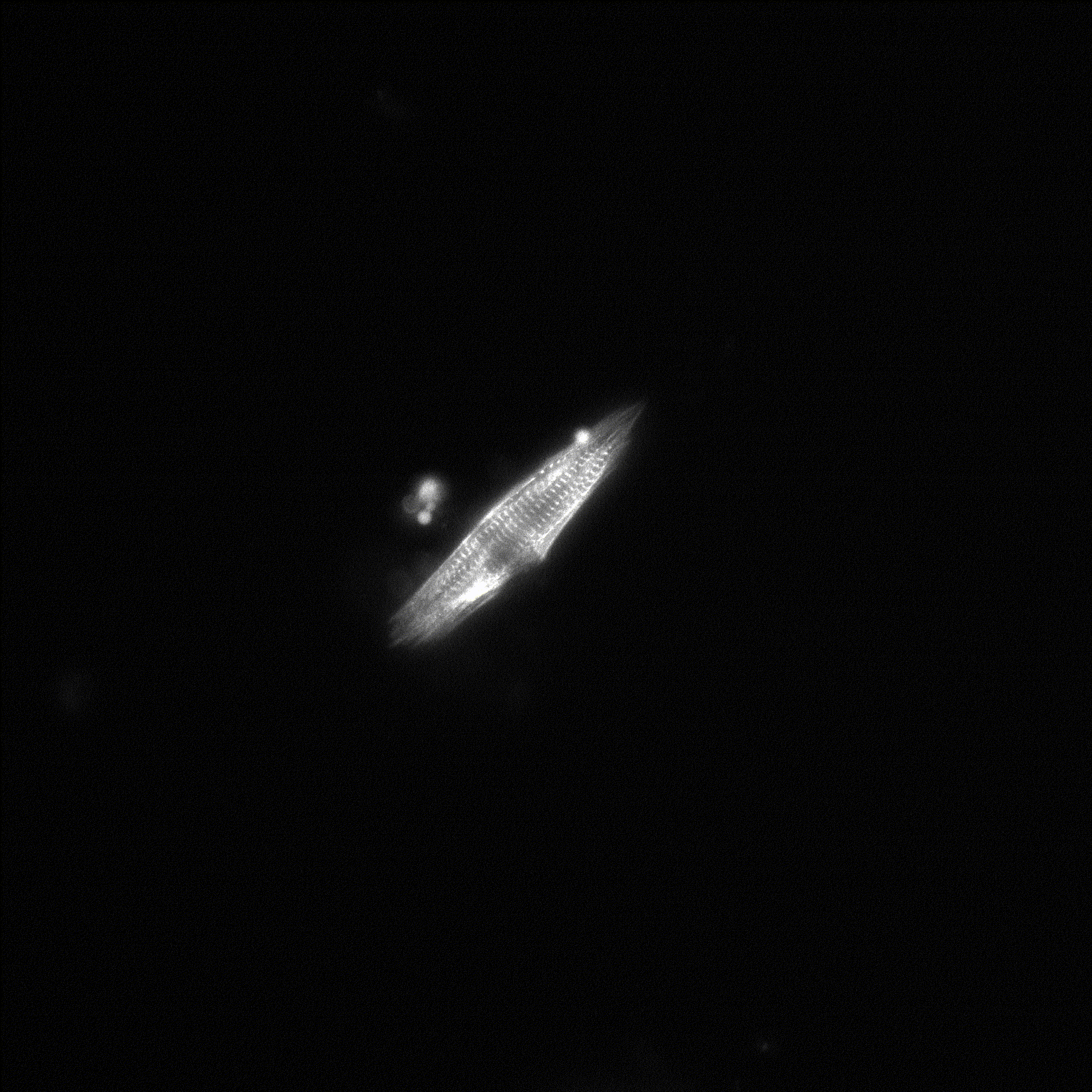

Supplement: cvaf037_Supplementary_Data [file cvaf037_supplementary_data.zip › TTN-AS_MS_Suppl_Video.gif]
